# Supplementary material for: Highly reinforced and degradable lignocellulose biocomposites by polymerization of new polyester oligomers
Source: Nat Commun. 2022 Sep 27;13:5666. doi: 10.1038/s41467-022-33283-z (PMC9515094; doi:10.1038/s41467-022-33283-z)
Supplement: Supplementary file 1 — Supplementary Information [file 41467_2022_33283_MOESM1_ESM.pdf]

Supplementary information

Highly reinforced and degradable lignocellulose biocomposites by polymerization of new polyester oligomers

Erfan Oliaei[1,2], Peter Olsén[2*], Tom Lindström[1], Lars A. Berglund[2*]

[1]*RISE Bioeconomy and health, Stockholm, Sweden (toml@kth.se)*

[2]*Wallenberg Wood Science Center, Department of Fibre and Polymer Technology, KTH Royal Institute of Technology, Stockholm, Sweden (oliaei@kth.se; *polsen@kth.se; *blund@kth.se)*

Table of content

Supplementary Discussion1- Effect of moisture on ring-opening polymerization of caprolactone

Supplementary Discussion2- Calculation method for end-groups ratio in the oligomers, and end-groups conversion for the polymers

Supplementary Equations 1-3

Supplementary Discussion3- Green chemistry metrics

Supplementary Equations 4-6

Synthesis details of caprolactone oligomers and their polymerization

Supplementary Tables 1-3

^{1}H NMR on the functional caprolactone-oligomers

Supplementary Figures 1-7

SEC curves for the optimization of the polymerization

Supplementary Figures 8-19

Thermal characteristics of the synthesized polymer (c-PCL) and the biocomposites

Supplementary Figures 20-21

Supplementary Table 4

Physicochemical and thermal properties of the biocomposites

Supplementary Figures 22-25

Supplementary Table 5

SEM study of cellulose fibers, MFLC, and the c-PCL biocomposites

Supplementary Figures 26-32

c-PCL biocomposites from hot-pressed wood fibers (HP-WF)

Supplementary Figures 33-35

Fiber distribution dimensions

Supplementary Figure 36

Molten caprolactone oligomer's viscosity

Supplementary Figure 37

Supplementary Discussion1- Effect of moisture on ring-opening polymerization of caprolactone

A typical cellulosic fiber in ambient conditions (RT, 50% RH) contains at least 5 wt% of moisture, making the maximum theoretical molecular weight around 8000 (calculated based on caprolactone $(\varepsilon\mathrm{CL})(n_{\varepsilon\mathrm{C}}\ /n_{H2O} = M_n)$ with only 5 wt% of cellulosic fiber.

Synthesis details of caprolactone oligomers and their polymerization

Supplementary Table 1. Synthesis of functional three-arm CL-oligomers

Entry	[I]:[M]:[SA][a]	Mn (Th)[b]	Mn (NMR)[c]	Mn (SEC)[d]	D (SEC)[d]	COOH:OH[e]	Yield (%)[f]	Oligomer[g]
**[1]**	[1]:[7]:[3]	1200	1500	700	2.4	2:1	31	Gly-PCL_7-SA_2
**[2]**	[1]:[14]:[3]	2000	1900	1500	2.7	1:1	75	Gly-PCL_{14}-$SA_{1.5}$
**[3]**	[1]:[21]:[3]	3500	2900	2100	3.5	1:1	82	Gly-PCL_{21}-$SA_{1.5}$

[a][I], [M], and [SA] are glycerol, monomer (caprolactone or CL), and succinic anhydride, respectively. [b]Calculated based on the feed ratio. [c]Determined by ^{1}H NMR. [d]Determined by SEC using $CHCl_3$ as eluent calibrated with polymethylmethacrylate (PMMA) standards having precise individual molecular weights. [e] Calculated by ^{1}H NMR according to supplementary equation 1. [f]Isolated yield. [g]Final composition in the oligomer as determined by ^{1}H NMR.

Supplementary Table 2. Polymerization data from CL-oligomers

Entry	Oligomer[a]	Mn[b] (Oligomer)	D[b] (Oligomer)	Catalyst (mol%)	Mn[c] (Polymer)	D[c] (Polymer)	Gel[d] (wt%)
**[1]**	Gly-PCL_7-SA_2	700	2.4	0	1600	6.0	1
**[2]**	Gly-PCL_{14}-$SA_{1.5}$	1500	2.7	0	3400	3.0	15
**[3]**	Gly-PCL_{21}-$SA_{1.5}$	2100	3.5	0	2700	4.0	3
**[4]**	Gly-PCL_7-SA_2	700	2.4	1.0	ND[e]	ND[e]	93
**[5]**	Gly-PCL_{14}-$SA_{1.5}$	1500	2.7	1.0	4100	4.3	77
**[6]**	Gly-PCL_{21}-$SA_{1.5}$	2100	3.5	1.0	10600	5.3	27
**[7]**	Gly-PCL_{14}-$SA_{1.5}$	1500	2.7	0.5	9300	13.8	43

[a]Starting composition of the oligomer as determined by ^{1}H NMR. [b]Determined by SEC using $CHCl_3$ as eluent with PMMA standards, [c]determined by SEC using $CHCl_3$ as eluent with polymethyl methacrylate standards on the extracted polymer, [d]remaining dry percentage after extraction in dimethylformamide: CH_2Cl_2 for 48h. [e]not detected.

Supplementary Table 3. Polymerization details for in-situ polyesterification for neat cross-linked polycaprolactone (c-PCL), and in MFLC and WF reinforcements. Note that reinforcement contents in c-PCL/MFLC41 and c-PCL/WF44 are similar (41 vs 44 wt%). Values in parentheses are standard deviations.

Sample	Gel (wt%)	Mn (dissolved c-PCL)	Con. OH	Con. COOH
c-PCL	43(2)	9,300	77	65
c-PCL/MFLC41	46(2)	7,000	48	38
c-PCL/WF44	75(4)	13,500	68	53

c-PCL from Gly-PCL_{14}-$SA_{1.5}$ with 0.5 mol% catalyst.

Supplementary Discussion2- Calculation method for end-groups ratio in the oligomers, and end-groups conversion for the polymers

The calculation of the end-groups ratio was performed in accordance with Supplementary Equation 1.

Supplementary Equation 1. COOH/OH

$$= \frac{i}{2 \times E} = \frac{\int_{2.55\,ppm}^{2.68\,ppm} i}{2 \times (\int_{3.58ppm}^{3.67ppm} E)}$$

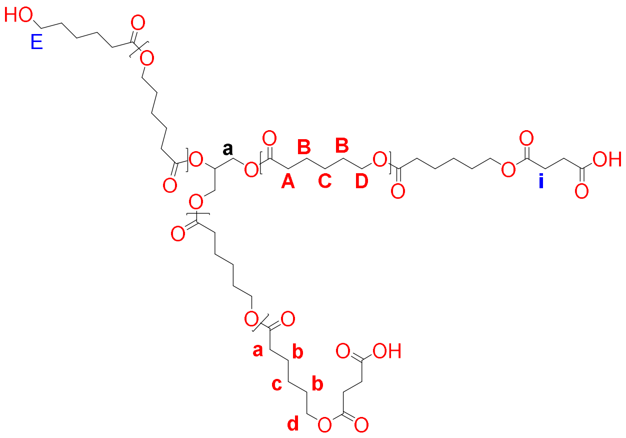

The calculation of end-groups conversion was performed according to Supplementary Equations 2 and 3.

Supplementary Equation 2. Con. COOH

$$= \left(\frac{ii}{i+ii}\right) \times 100 = (\frac{\int_{2.61\,ppm}^{2.64\,ppm} ii}{(\int_{2.64ppm}^{2.70ppm} i + \int_{2.61ppm}^{2.64ppm} ii)}) \times 100)$$

Supplementary Equation 3. Con. OH

$$= \frac{COOH}{OH}(start) \times \left(\frac{E \times 2}{i + ii}\right) \times 100 = (\frac{2 \times \left(\int_{3.58\,ppm}^{2.67\,ppm} E\right)}{(\int_{2.64ppm}^{2.70ppm} i + \int_{2.61ppm}^{2.64ppm} ii)}) \times 100$$

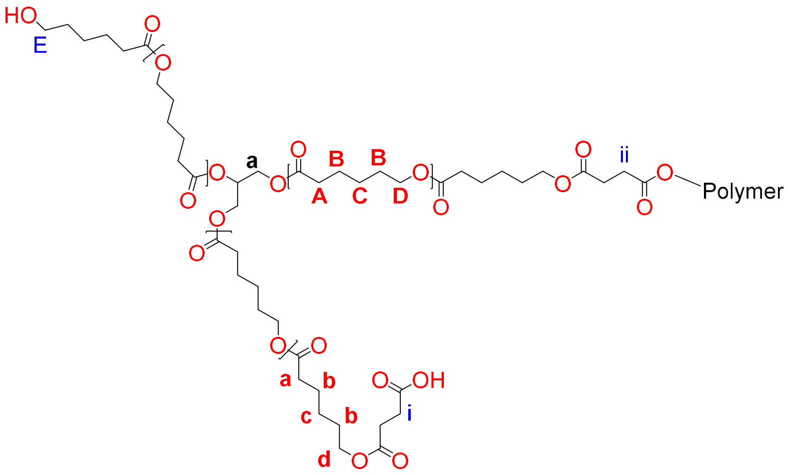

Supplementary Discussion3- Green chemistry metrics

Atom economy:

Trost's atom economy is a metric to show the reaction efficiency and related to the number of atoms of reactants appearing in the product[1,2], calculated as

Supplementary Equation 4.

$$Atom\ economy\ = \frac{molecular\ mass\ of\ desired\ product}{molecular\ masses\ of\ reactants}\text{; (In the stoichiometric equation)}$$

We have 3 different reactions: ROP of ε-caprolactone, end-capping, and polyesterification reactions.

ROP reaction:

$C_3H_8O_3$ + 14 $C_6H_{10}O_2$ → $C_{87}H_{148}O_{31}$

End-capping reaction:

$C_{87}H_{148}O_{31}$ + $C_4H_4O_3$ → $C_{91}H_{152}O_{34}$ (Oligomer with 1 COOH functional group, OligoA)

$C_{87}H_{148}O_{31}$ + 2 $C_4H_4O_3$ → $C_{95}H_{156}O_{37}$ (Oligomer with 2 COOH functional group, OligoB)

Atom economy of ROP and end-capping for the functionally balanced oligomer (actually a mixture of OligoA and OligoB)

OligoA= 1788 / (92+14*114+100) = 1788 =100%

OligoB= 1788 / (92+14*114+2*100) = 1888 =100%

Polyesterification reaction:

ROOH + R´OH → ROOR´ + H_2O (ROOH and R´OH are oligomers or product of their esterification)

Atom economy of polyesterification = (molecular mass of ROOR´) / (sum of molecular masses of ROOH + R´OH) > (1788*2-18)/(1788*2) > 99.5%

In polyesterification, water molecule is released. Esterification of smallest oligomers make a di-oligomer with the least possible atom economy of polyesterification, since the product has the least possible mass (relative to mass of undesired product, i.e. water).

Note that desired product is c-PCL, both the gel and non-gel fractions. Therefore, atom economy of the whole series of reactions from oligomer synthesis to polyesterification is > 99.5%.

Reaction mass efficiency:

Reaction mass efficiency is defined as the ratio of the actual mass of the desired product to the total mass of all reactants employed[3]. It considers both atom economy and chemical yield (e.g. excess reagents)[1,3].

Supplementary Equation 5.

$$Reaction\ mass\ efficiency\ = \frac{mass\ of\ desired\ product}{masses\ of\ reactants}\text{;(In practice)}$$

Reaction mass efficiency of ROP and end-capping = 1500 / (92+114*14+100*3) = 75%

Reaction mass efficiency of polyesterification=100%

The reaction mass efficiency of the whole series of reactions from oligomer preparation to polyesterification is 75% (75%*100%), and for only the in-situ polymerization/curing part is 100%.

Sheldon's environmental factor (E factor):

Sheldon's E-factor of a process is defined as Supplementary Equation 6.

$$\mathrm{E-factor} = \frac{mass\ of\ waste}{mass\ of\ product}$$

E factor for the synthesis of the selected oligomers (Gly-PCL_{14}-$SA_{1.5}$ with 0.5 mol% catalyst) is calculated:

Waste [30 ml of dioxane (30.9 g)+ 0.5*methanesulfonic acid (0.5*0.07 g)+ 0.5*succinic anhydride (0.5*1.55 g)] / product [caprolactone (8.3 g)*yield(~0.75)] => Sheldon's E factor (30.9+0.03+0.77 g)/(6.22 g) = 5.1

Note that in the scaled-up synthesis of oligomers, the solvent is not discarded but recycled. Also, the yield would be much higher than75% since oligomer isolation procedure will be optimized. Therefore, Sheldon's E factor would be much lower than 5.

All the waste in the polymerization system (from oligomers) is related to ethanol and acetone losses in solvent exchange and impregnation. Each time solvent equal to 2.5 times the weight of the product is used for solvent exchange, and acetone equal to 5 times the weight of the product is used for impregnation. One time, ethanol and 3 times acetone were exchanged, and then fresh acetone was used for impregnation. Hence, Sheldon's E factor for the in-situ polymerization part is approximately 15,

E-factor = 1*2.5+3*2.5+1*5 = 15

Note here the aim is to create "model" biocomposites with homogeneous polymer distribution and low void content. Hence, solvent exchange and solvent-assisted impregnation are used to serve this purpose. However, for large-scale production, the use of solvents is not needed (at 140 °C the oligomers are liquid and can diffuse to fiber networks). Therefore, from a large-scale perspective, Sheldon's E factor for the polymerization part is almost zero (near ideal) since everything that remains in the system is desired, and there is no loss or byproducts. However, quality may, to some extent, be compromised.

^{1}H NMR on the functional caprolactone-oligomers

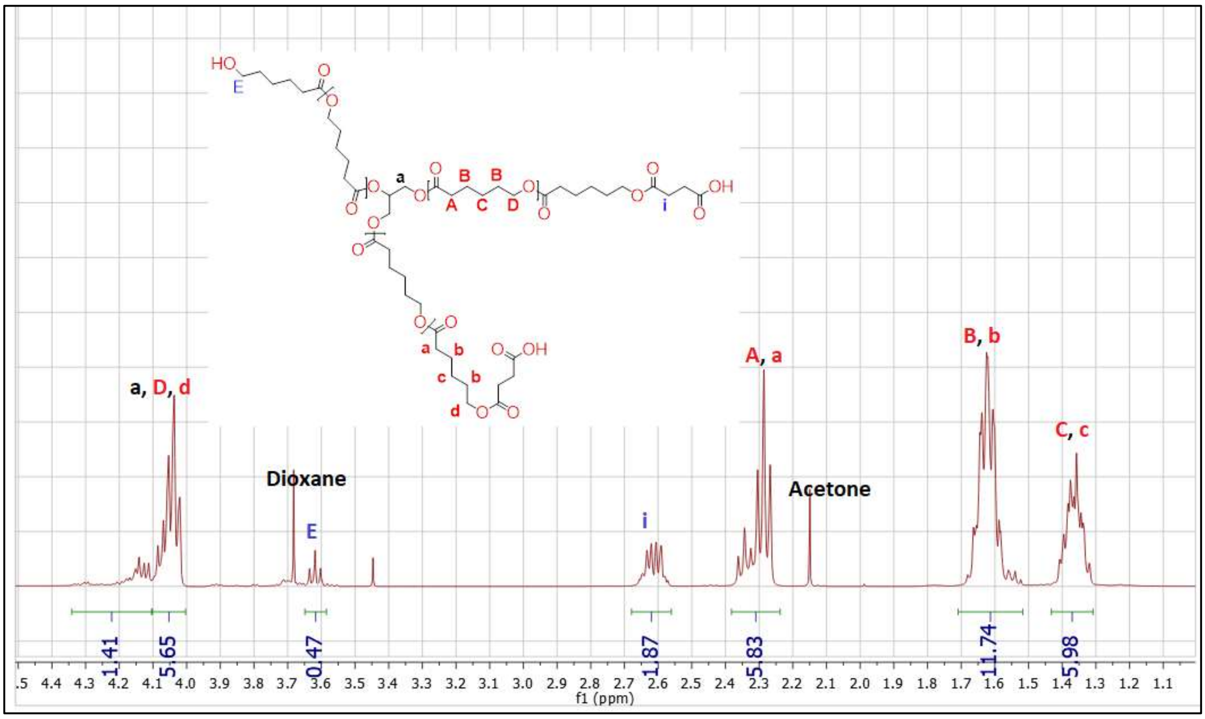

Supplementary Figure 1. ^{1}H NMR on the purified oligomer Gly-PCL$_7$-SA$_2$. The end-group ratio (COOH/OH) was calculated according to Supplementary Equation 1.

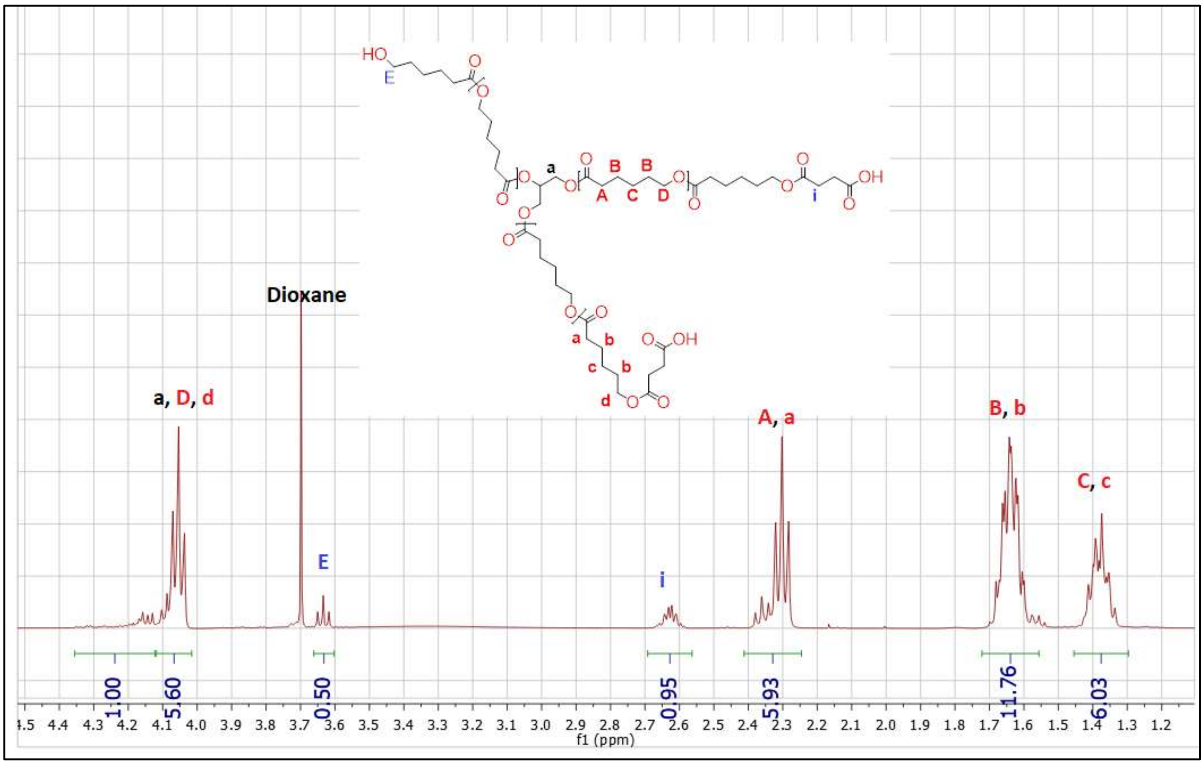

Supplementary Figure 2. ^{1}H NMR on the purified oligomer Gly-PCL$_{14}$-SA$_{1.5}$. The end-group ratio (COOH/OH) was calculated according to Supplementary Equation 1.

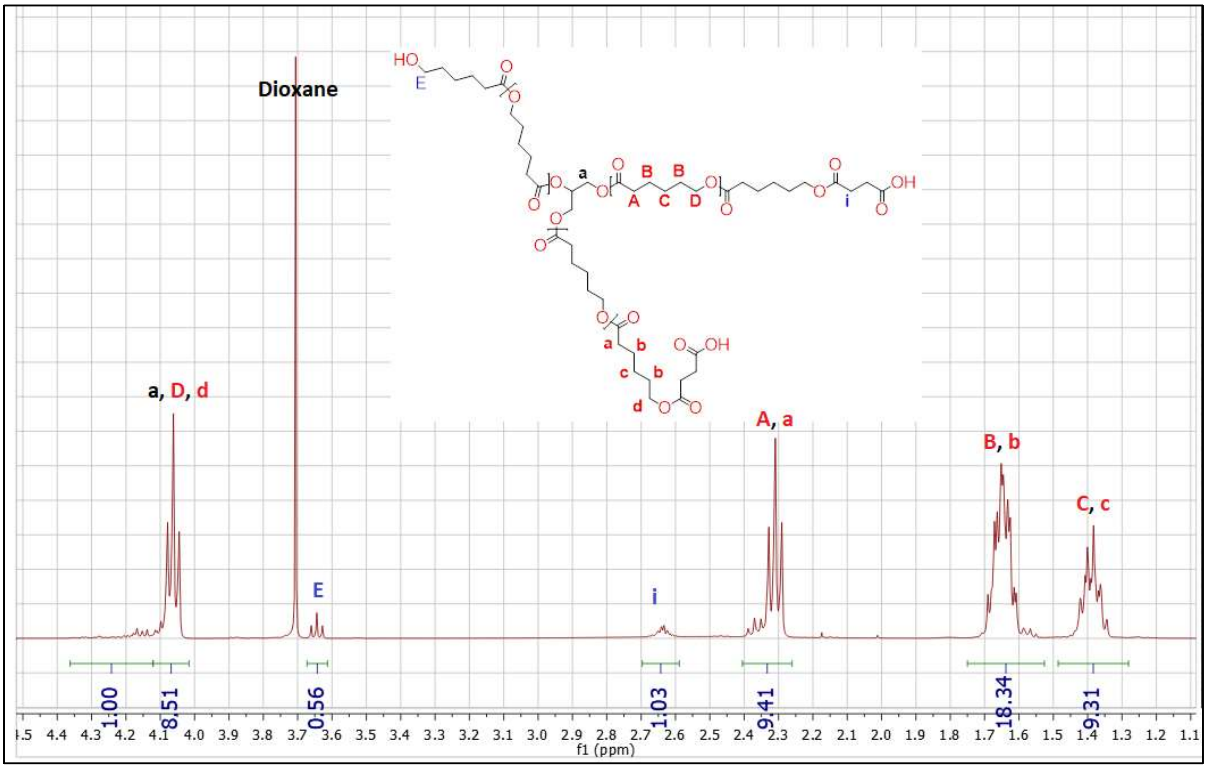

Supplementary Figure 3. ^{1}H NMR on the purified oligomer Gly-PCL$_{21}$-SA$_{1.5}$. The end-group ratio (COOH/OH) was calculated according to Supplementary Equation 1.

^{1}H NMR on the extracted polymer after in-situ condensation polymerization

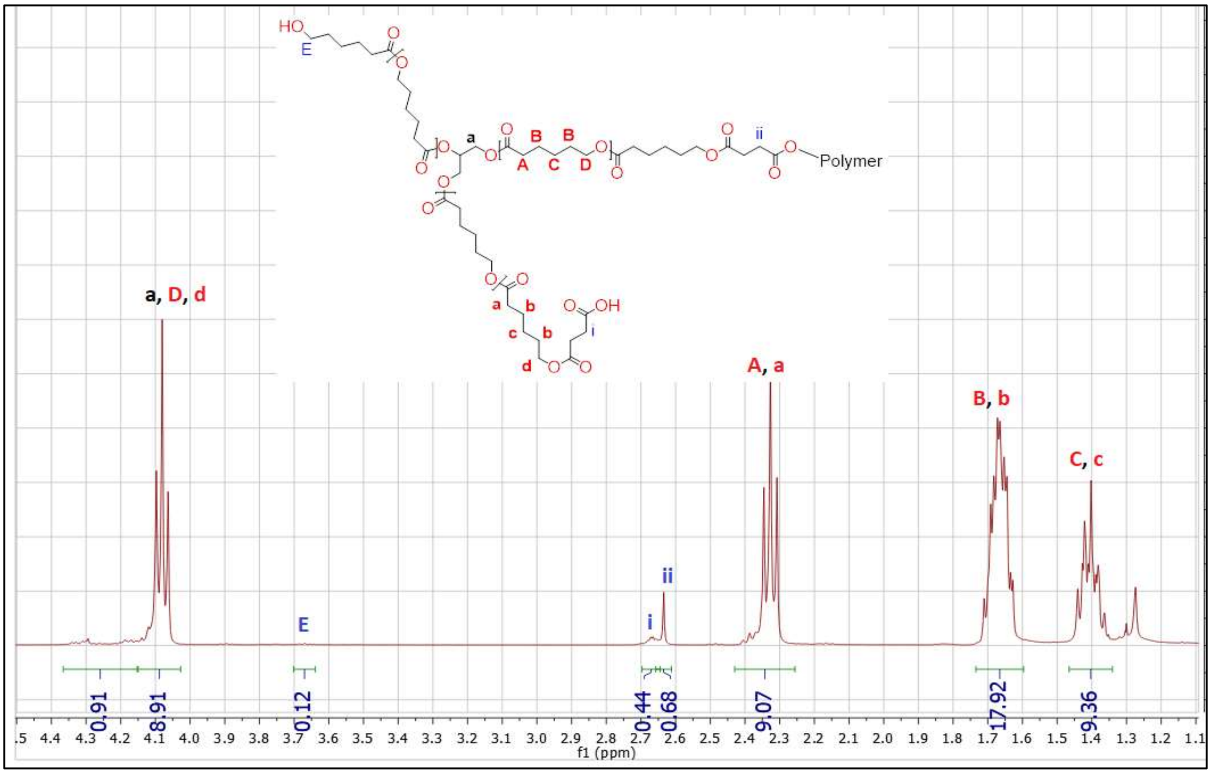

Supplementary Figure 4. ^{1}H NMR on the extracted polymer (57 wt%) after in-situ polycondensation of Gly-PCL$_{14}$-SA$_{1.5}$ at 140 °C for 14 h catalyzed with 0.5 mol% Sn(Oct)$_2$. End-group conversion, COOH 65 %, and OH 77 % were calculated according to Supplementary Equations 2 and 3.

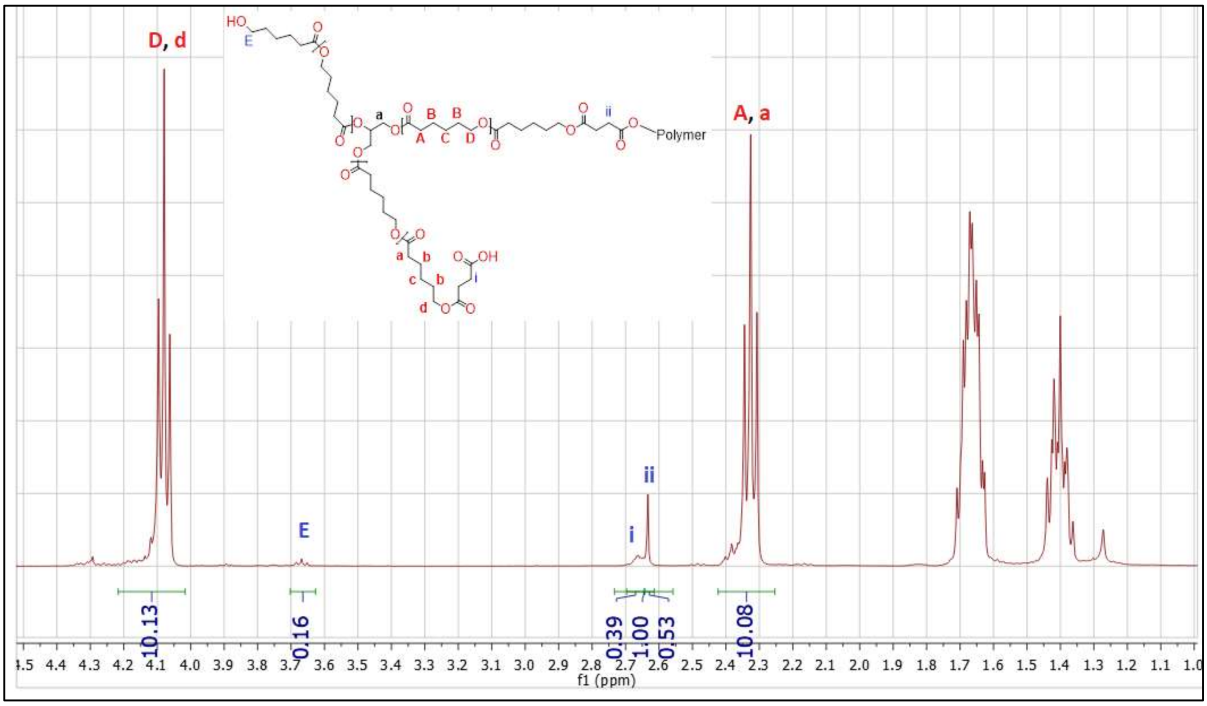

Supplementary Figure 5. ^{1}H NMR on the extracted polymer (25 wt%) after in-situ polycondensation of Gly-PCL_{14}-$SA_{1.5}$ around 44 wt% WF at 140 °C for 14 h catalyzed with 0.5 mol% $Sn(Oct)_2$. End-group conversion, COOH 53 %, and OH 68 % were calculated according to Supplementary Equations 2 and 3.

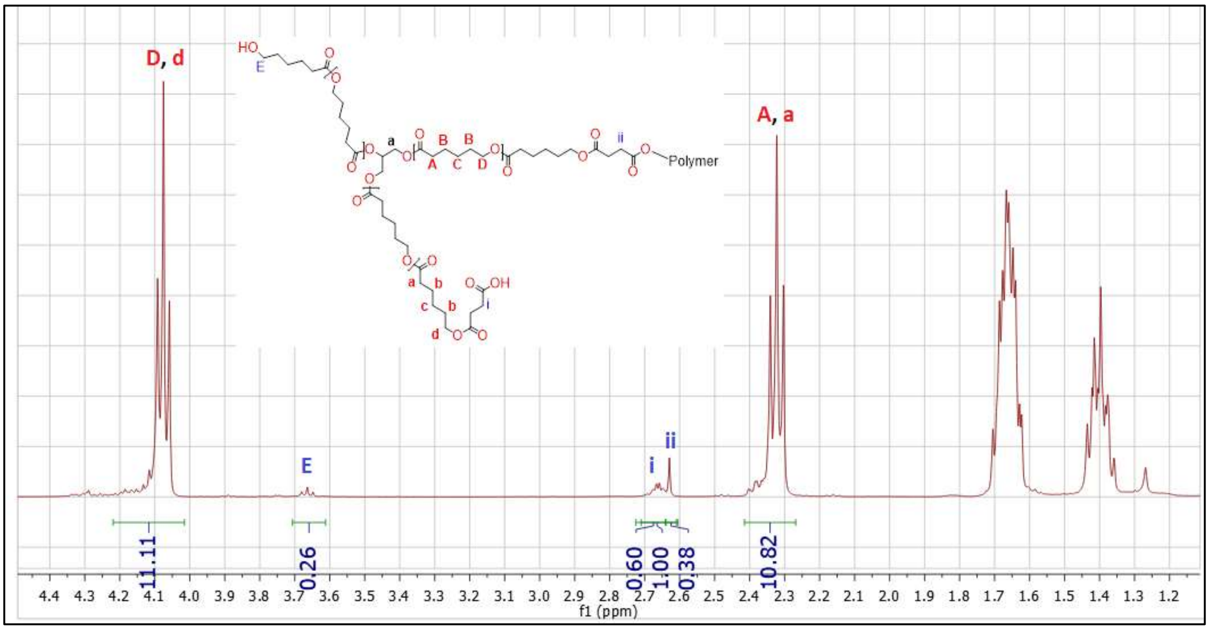

Supplementary Figure 6. ^{1}H NMR on the extracted polymer (54 wt%) after in-situ polycondensation of Gly-PCL_{14}-$SA_{1.5}$ around 41 wt% MFLC at 140 °C for 14 h catalyzed with 0.5 mol% $Sn(Oct)_2$. End-group conversion, COOH 38 %, and OH 48 % were calculated according to Supplementary Equations 2 and 3.

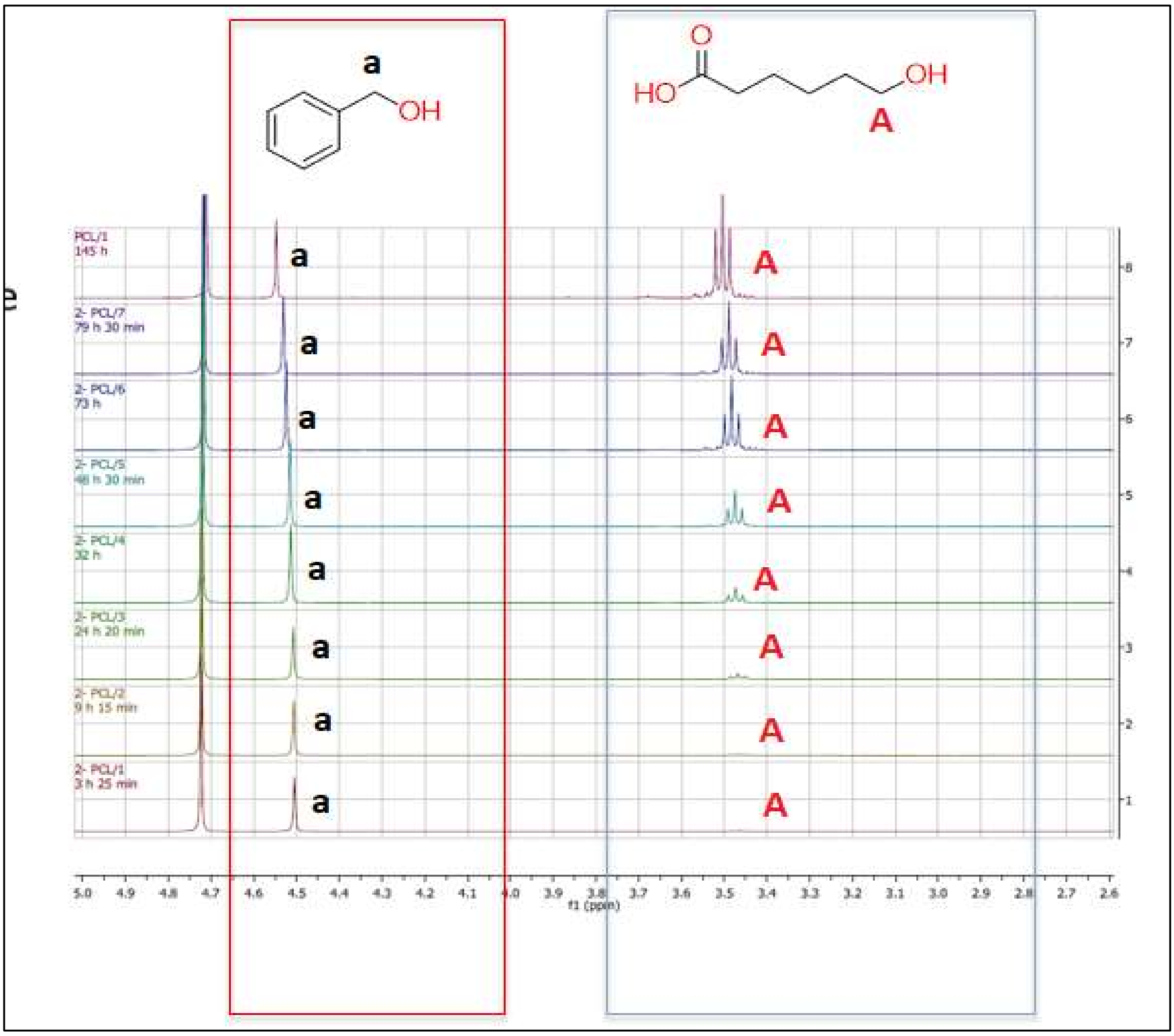

Supplementary Figure 7. ^{1}H NMR over time for accelerated degradation of the c-PCL homopolymer under alkaline conditions (pH 12). BnOH was used as an internal standard. The degradation was assessed by following the increase in end-groups.

SEC curves for the optimization of the polymerization

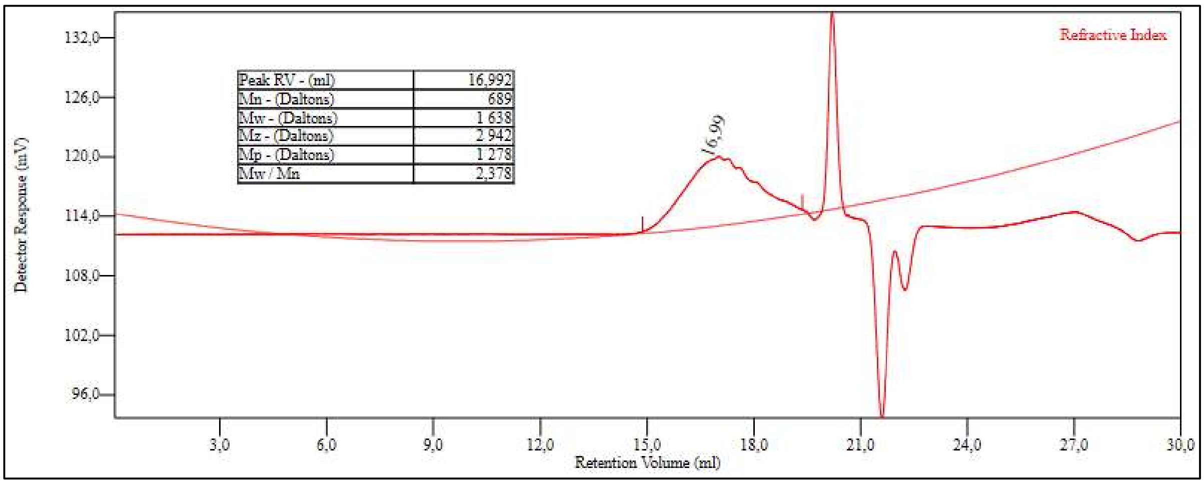

Supplementary Figure 8. SEC traces for Gly-PCL_7-SA_2. The SEC system used $CHCl_3$ as eluent with toluene as the internal standard, calibrated with narrow PMMA standards.

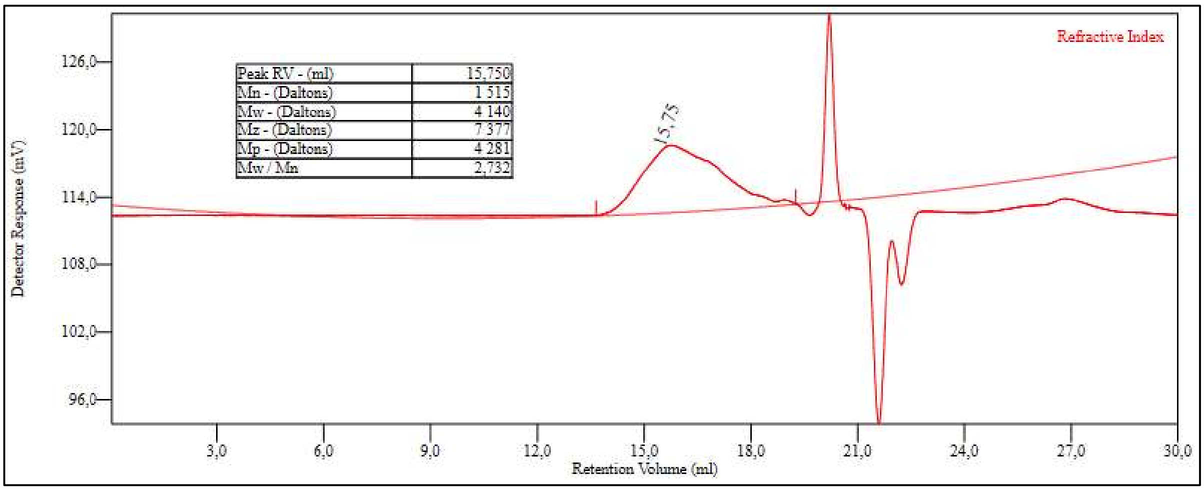

Supplementary Figure 9. SEC traces for Gly-PCL_{14}-$SA_{1.5}$. The SEC system used $CHCl_3$ as eluent with toluene as the internal standard, calibrated with narrow PMMA standards.

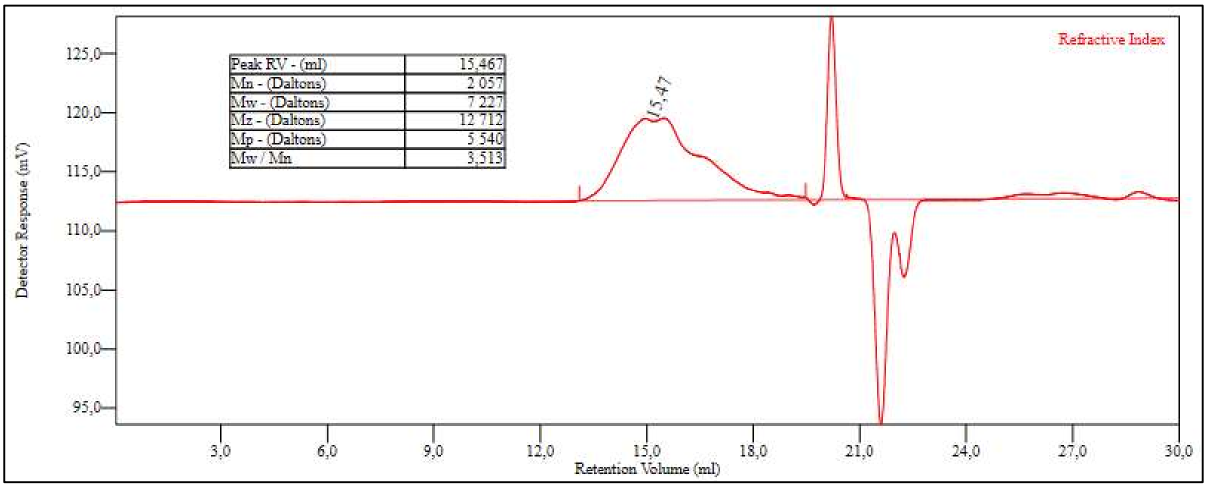

Supplementary Figure 10. SEC traces for Gly-PCL_{21}-$SA_{1.5}$. The SEC system used $CHCl_3$ as eluent with toluene as the internal standard. The system was calibrated with narrow PMMA standards.

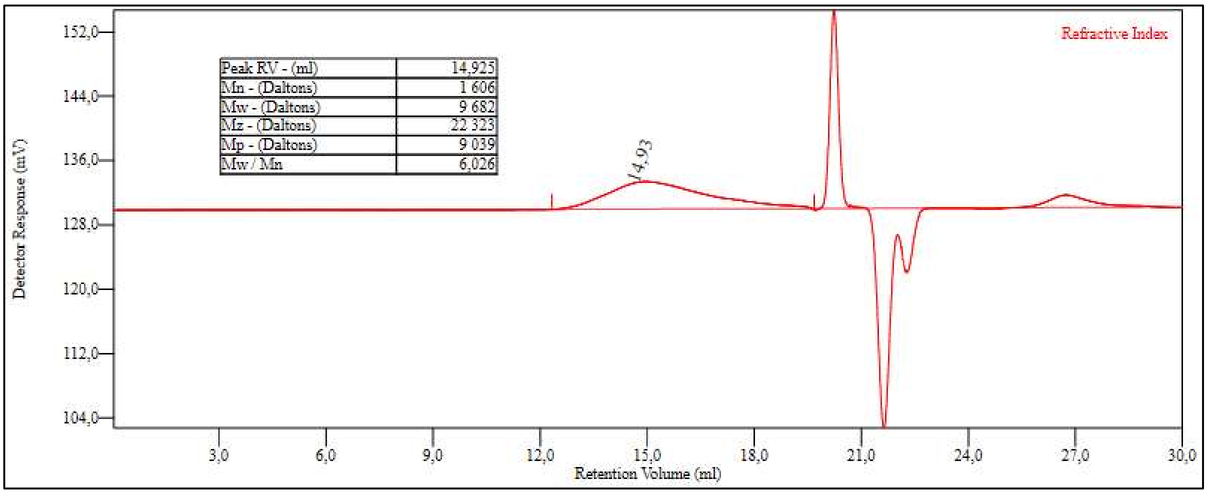

Supplementary Figure 11. SEC traces for extracted polymer after polycondensation of Gly-PCL_7-SA_2 at 140 °C for 14 h. The SEC system used $CHCl_3$ as eluent with toluene as the internal standard, calibrated with narrow PMMA standards.

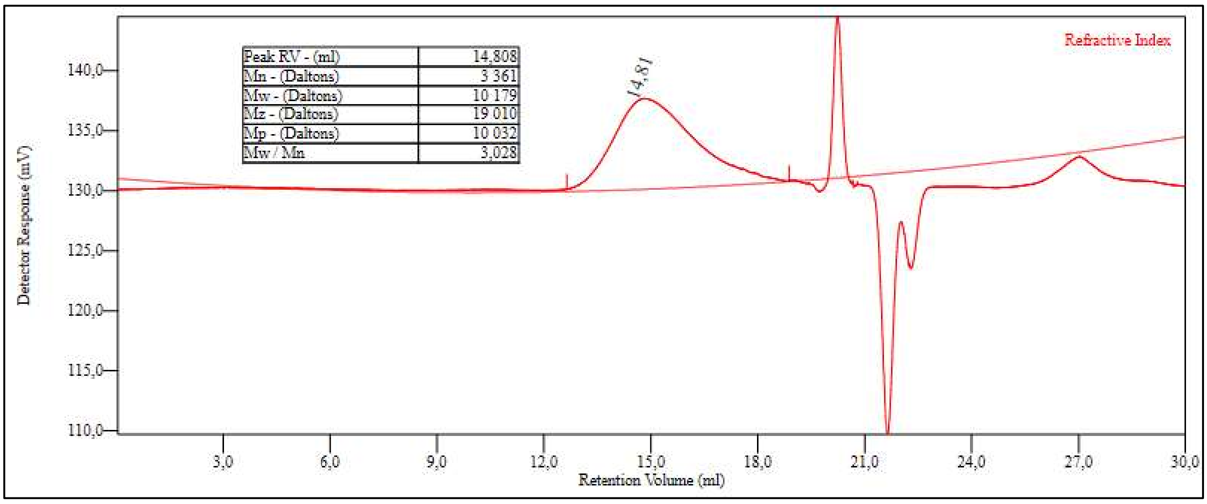

Supplementary Figure 12. SEC traces for extracted polymer after polycondensation of Gly-PCL$_{14}$-SA$_{1.5}$ at 140 °C for 14 h. The SEC system used $CHCl_3$ as eluent with toluene as the internal standard, calibrated with narrow PMMA standards.

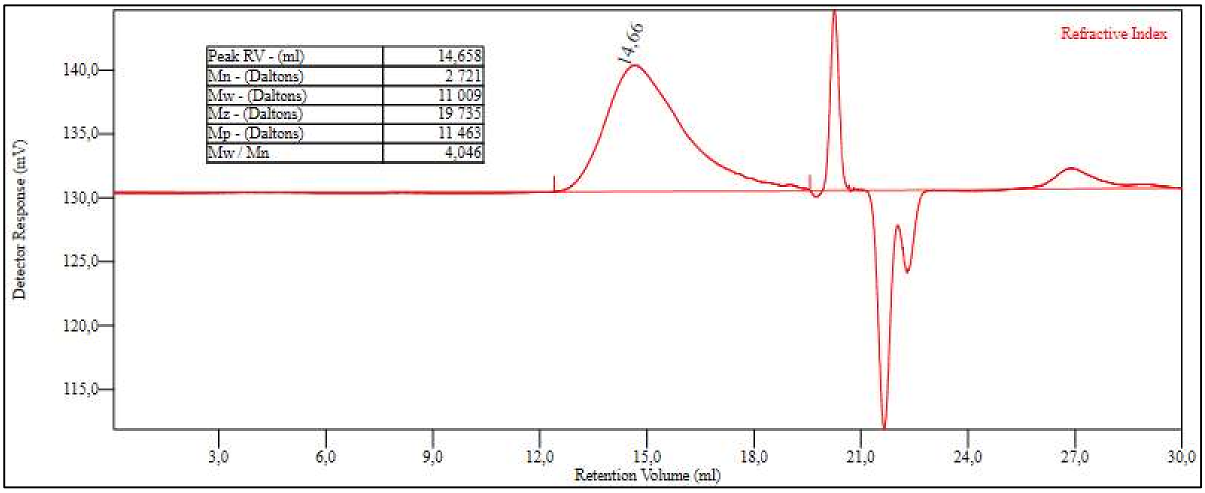

Supplementary Figure 13. SEC traces for extracted polymer after polycondensation of Gly-PCL$_{21}$-SA$_{1.5}$ at 140 °C for 14 h. The SEC system used $CHCl_3$ as eluent with toluene as the internal standard, calibrated with narrow PMMA standards.

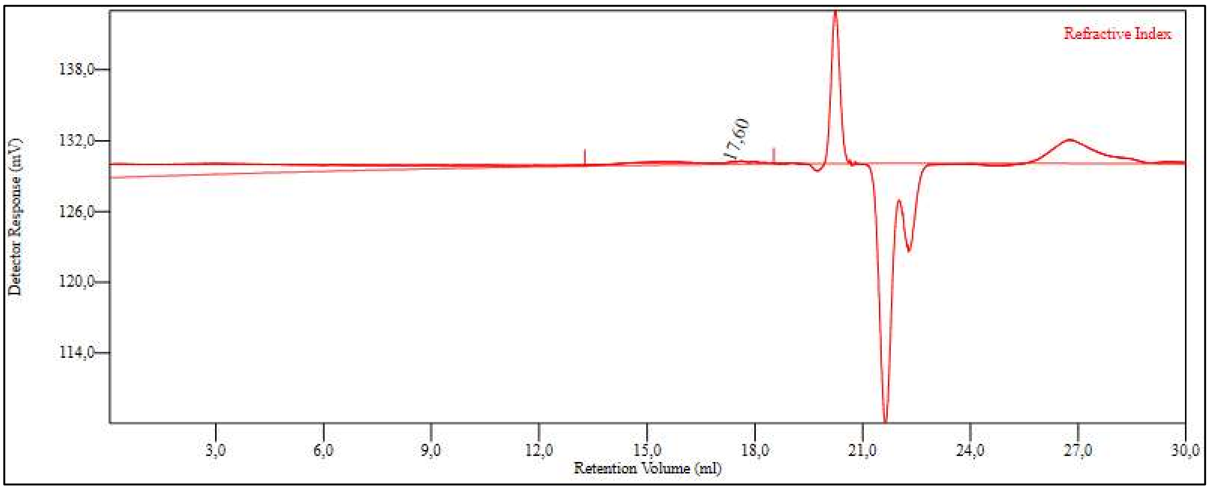

Supplementary Figure 14. SEC traces for extracted polymer after polycondensation of Gly-PCL$_7$-SA$_2$ at 140 °C for 14 h, catalyzed with 1 mol % Sn(Oct)$_2$. The SEC system used $CHCl_3$ as eluent with toluene as the internal standard, calibrated with narrow PMMA standards.

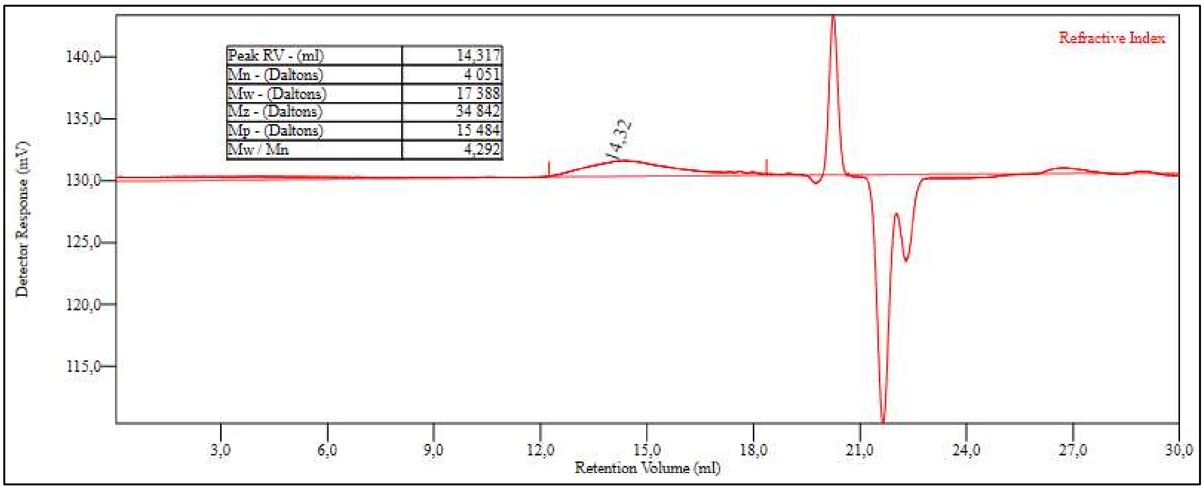

Supplementary Figure 15. SEC traces for extracted polymer after polycondensation of Gly-PCL_{14}-$SA_{1.5}$ at 140 °C for 14 h, catalyzed with 1 mol % $Sn(Oct)_2$. The SEC system used $CHCl_3$ as eluent with toluene as the internal standard, calibrated with narrow PMMA standards.

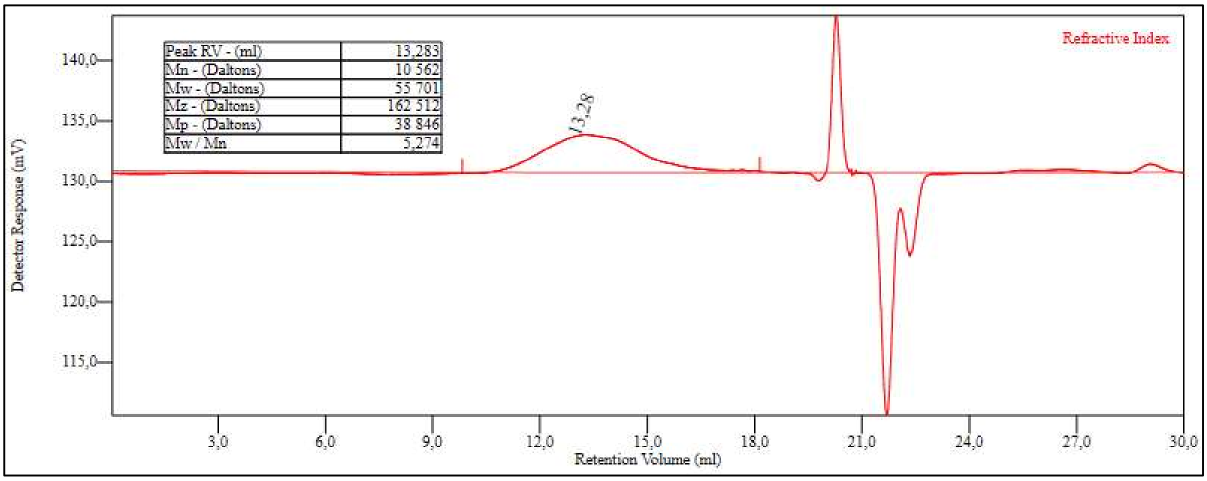

Supplementary Figure 16. SEC traces for extracted polymer after polycondensation of Gly-PCL_{21}-SA_2 at 140 °C for 14 h, catalyzed with 1 mol % $Sn(Oct)_2$. The SEC system used $CHCl_3$ as eluent with toluene as the internal standard, calibrated with narrow PMMA standards.

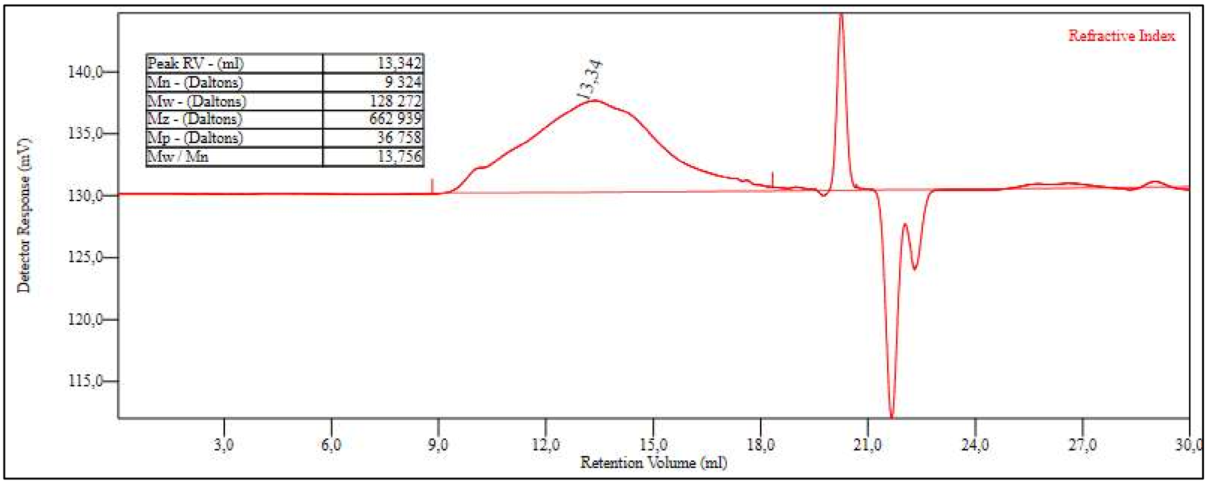

Supplementary Figure 17 SEC traces for extracted polymer after polycondensation of Gly-PCL_{14}-$SA_{1.5}$ at 140 °C for 14 h, catalyzed with 0.5 mol % $Sn(Oct)_2$. The SEC system used $CHCl_3$ as eluent with toluene as the internal standard, calibrated with narrow PMMA standards.

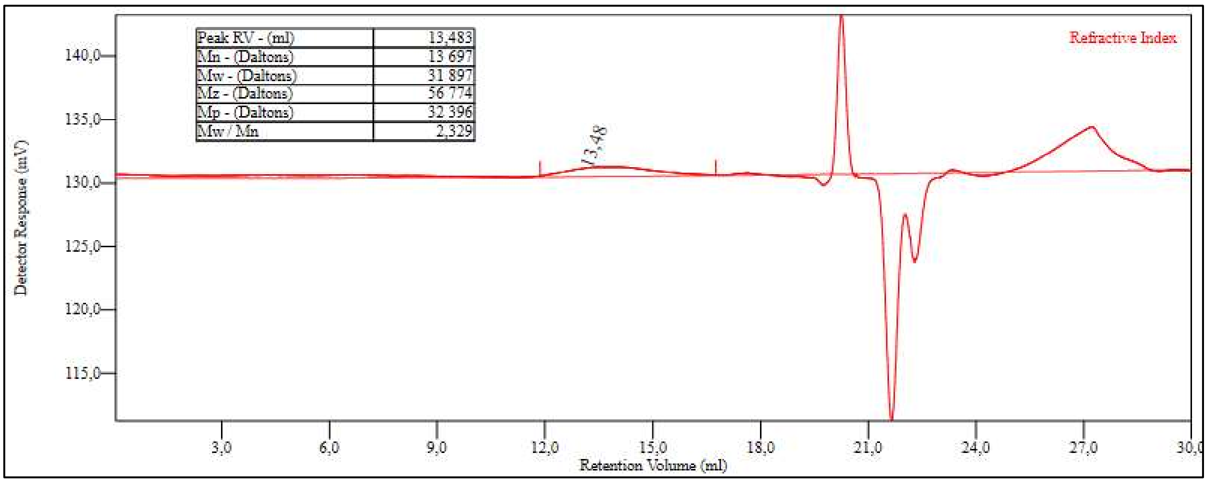

Supplementary Figure 18 SEC traces for extracted polymer after for polycondensation of Gly-PCL_{14}-$SA_{1.5}$ at 140 °C for 14 h, catalyzed with 0.5 mol % $Sn(Oct)_2$ in the presence of WF 44 wt %. The SEC system used $CHCl_3$ as eluent with toluene as the internal standard, calibrated with narrow PMMA standards.

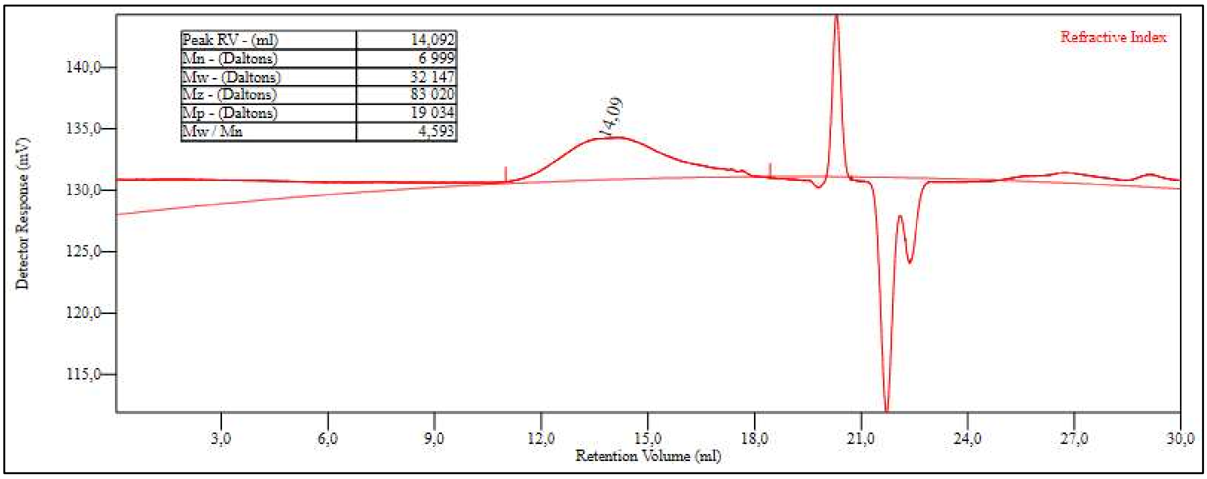

Supplementary Figure 19 SEC traces for extracted polymer after for polycondensation of Gly-PCL_{14}-$SA_{1.5}$ at 140 °C for 14 h, catalyzed with 0.5 mol % $Sn(Oct)_2$ in the presence of MFLC 41 wt%. The SEC system used $CHCl_3$ as eluent with toluene as the internal standard, calibrated with narrow PMMA standards.

Thermal characteristics of the synthesized polymer (c-PCL) and the biocomposites

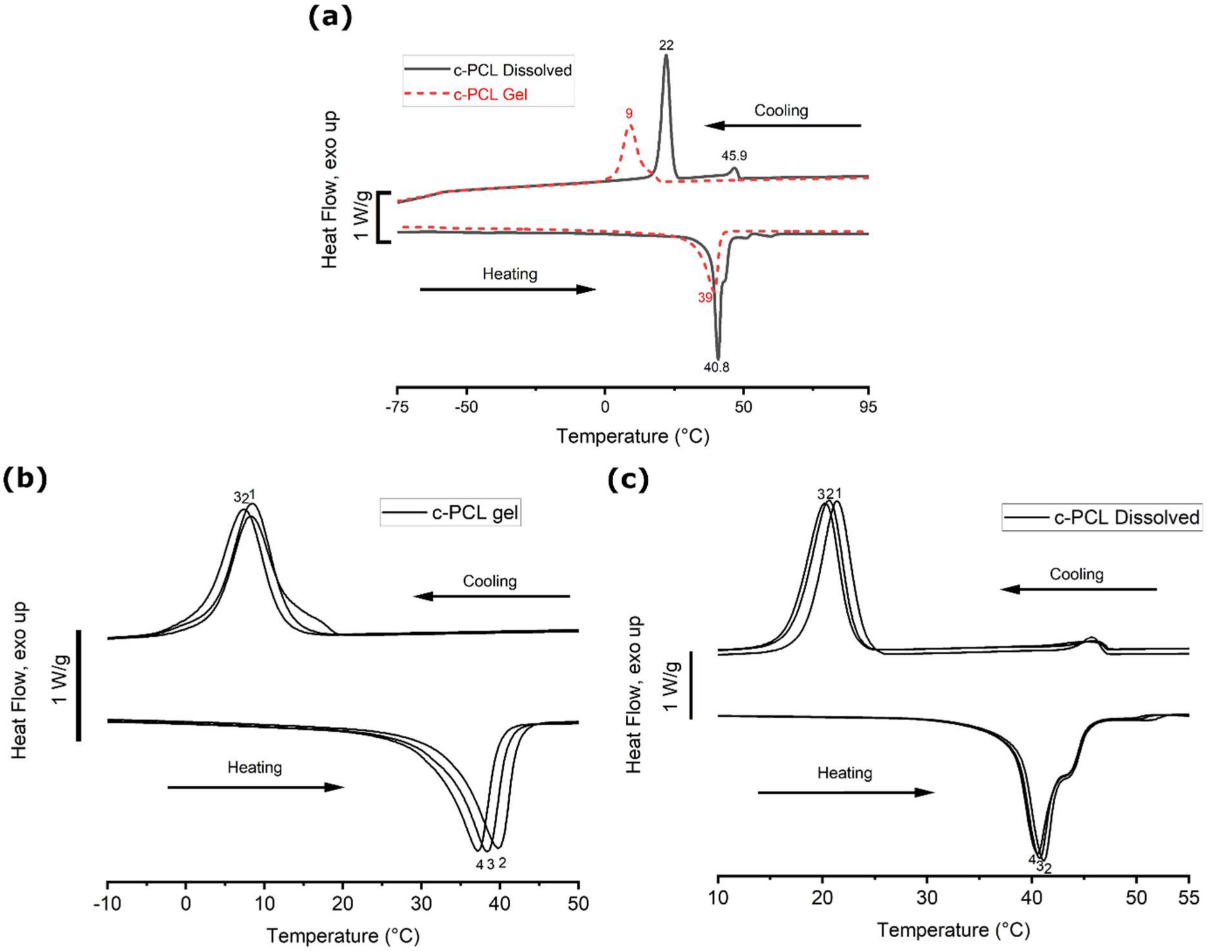

Supplementary Figure 20 (a) DSC curves of the second heating cycle and cooling cycle of dissolved and gel parts of c-PCL. Multiple heating and cooling cycles of **(b)** gel and **(C)** dissolved c-PCL, with 2 min isothermal 200 °C isothermal intervals between each cycle (curves are labeled with heating or cooling cycle number).

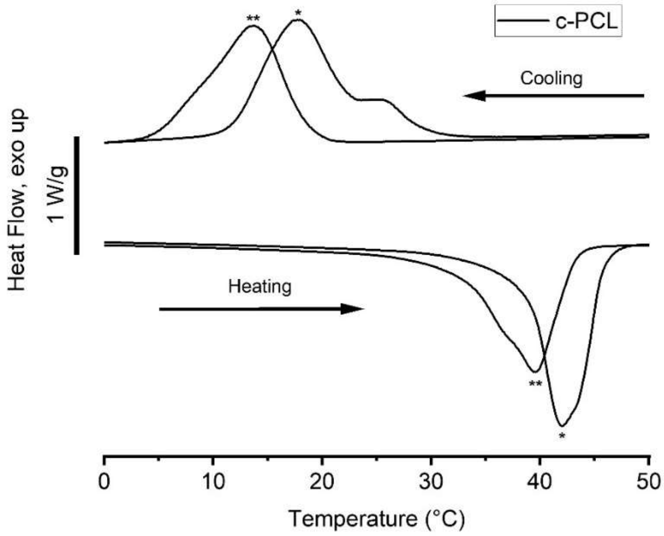

Supplementary Figure 21 Heating and cooling DSC curves of c-PCL after thermal history removal (*) and after 20 min remaining in 200 °C (**).

The shift of cooling peak toward lower temperatures upon thermal treatment at high temperatures was observed for the whole c-PCL (Supplementary Figure 21), the dissolved and undissolved part of it (Supplementary Figure 20). This shows that the system is still reactive, and it is likely that c-PCL further crosslinks at higher temperatures.

Supplementary Table 4 Thermal characteristics of dissolved and gel c-PCL in DMF/DCM (70/30) at room temperature for 48h. See Supplementary Figure 1 as well.

**Sample**	**ΔH_c (J/g)**	**ΔH_m (J/g)**	**Degree of crystallinity (%)**
Gel c-PCL	10.3	9.1	40.2
Dissolved PCL	13.6	11.8	51.9

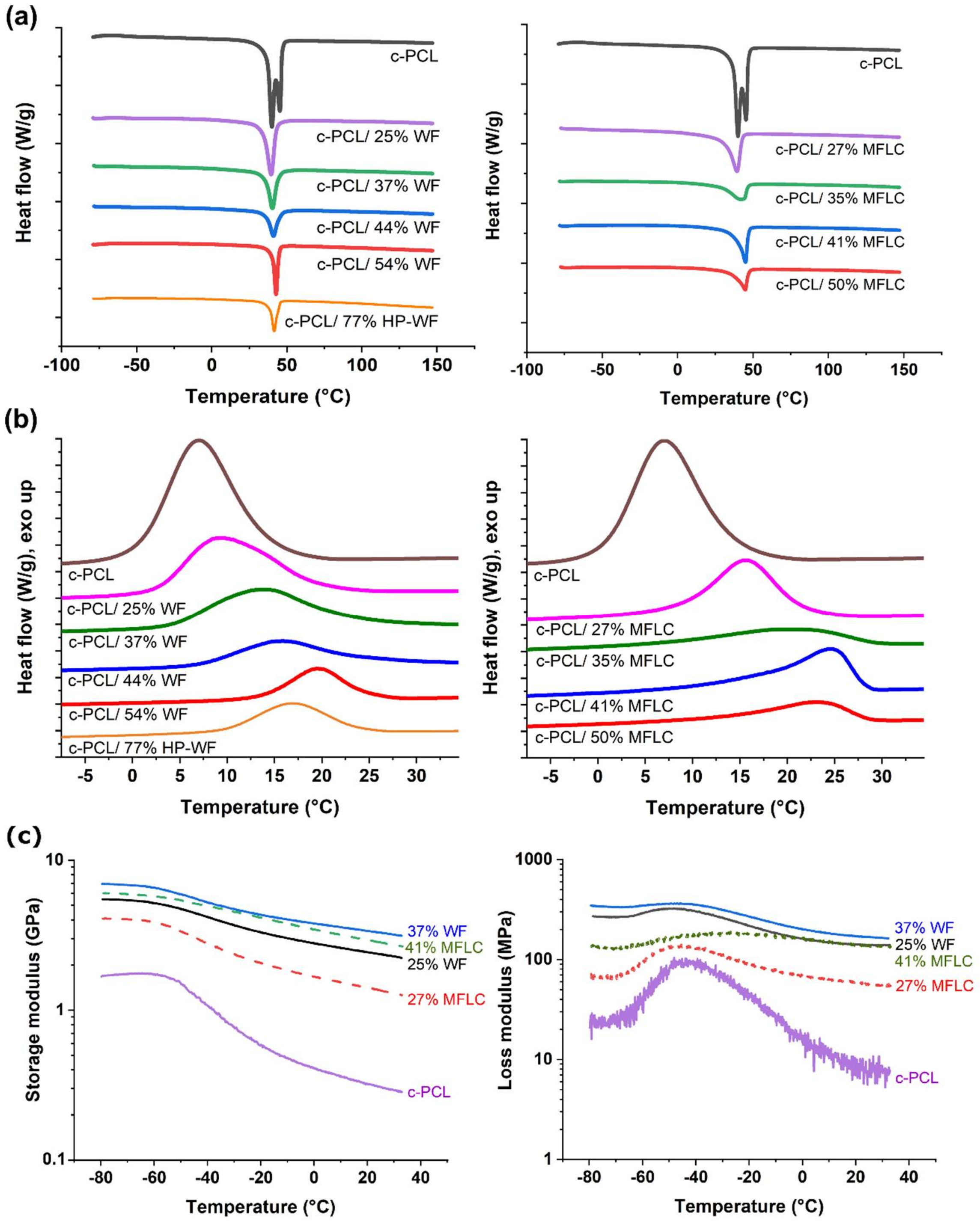

Supplementary Figure 22 Differential scanning calorimetry and dynamic mechanical analysis data of the biocomposites **(a)** Curves from the second heating cycle of wood fibers (WF) and microfibrillated lignocellulose (MFLC) biocomposites. **(b)** Curves from the first cooling cycle of WF and MFLC biocomposites. **(c)** Storage and loss moduli of WF and MFLC reinforced cross-linked polycaprolactone (c-PCL) biocomposites.

Physicochemical and thermal properties of the biocomposites

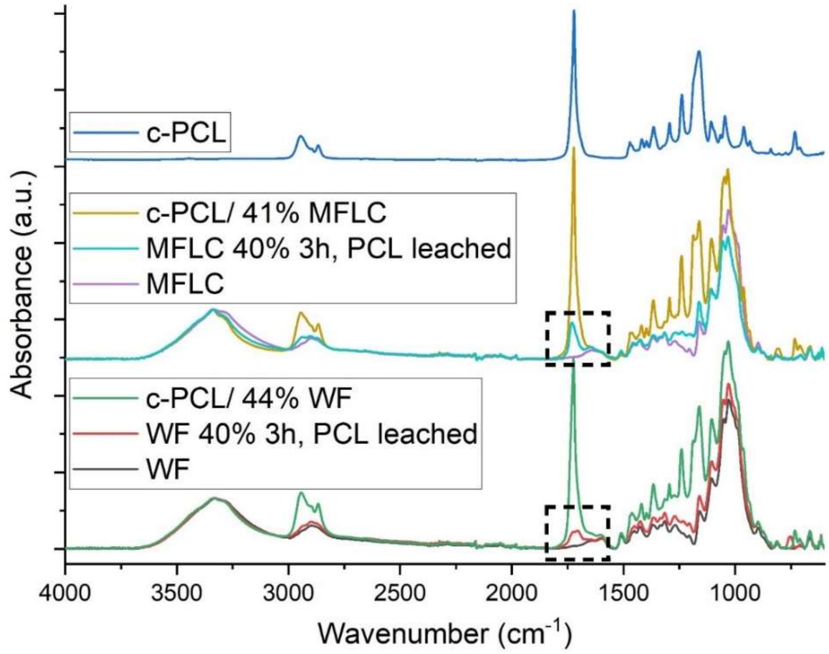

Supplementary Figure 23 FTIR spectra of the biocomposites (14 h reaction) and composites after 3 h synthesis and c-PCL extraction, showing covalent linkages of c-PCL to the reinforcement.

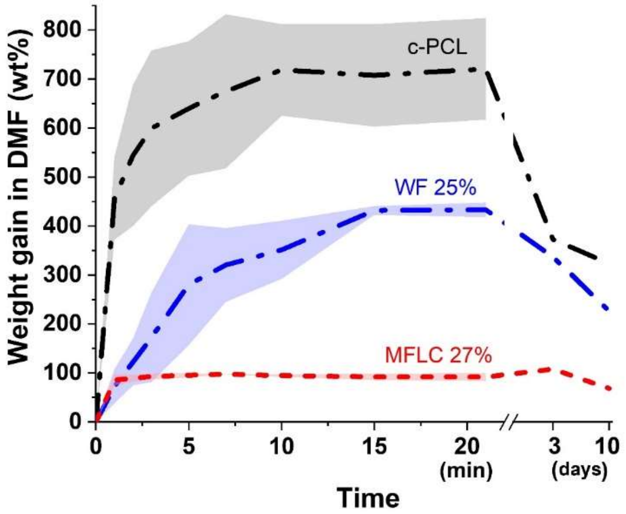

Supplementary Figure 24 Weight gain of the materials in DMF (wt %, normalized to c-PCL content) over time, related to swelling and deswelling (polymer dissolution).

Supplementary Table 5. Thermal characteristics of c-PCL biocomposites extracted from DMA and DSC data after thermal history removal, see Supplementary Figure 22.

Sample	T_g (◦C)*	T_m (◦C)	Normalized ΔH_m (J/g)	Degree of crystallinity (%)	T_c (◦C)	Normalized ΔH_c (J/g)
**c-PCL**	-43.8	40.1 (& 45.1)	67.2	49.4	7.1	20.7
**25% WF**	-	39.7	53.4	39.1	9.3	16.7
**37% WF**	-43.6	40.6	47.4	34.9	13.9	15.4
**44% WF**	-	41.1	38.4	28.0	15.9	10.2
**54% WF**	-47.7	42.8	42.0	30.8	19.6	10.2

**c-PCL/77% HP-WF**	-	41.5	73.2	53.6	16.9	23.0
**27% MFLC**	-45.4	39.7	46.2	33.8	15.8	14.1
**35% MFLC**	-	42.5	33.6	24.6	20.5	8.2
**41% MFLC**	-26.4	45.4	48.0	35.0	24.8	14.1
**50% MFLC**	-	45.0	38.4	28.0	23.0	9.4

*From DMA**.** The calculated degree of crystallinity of c-PCL, assuming melting enthalpy of 136.4 J/g for 100% crystalline PCL.

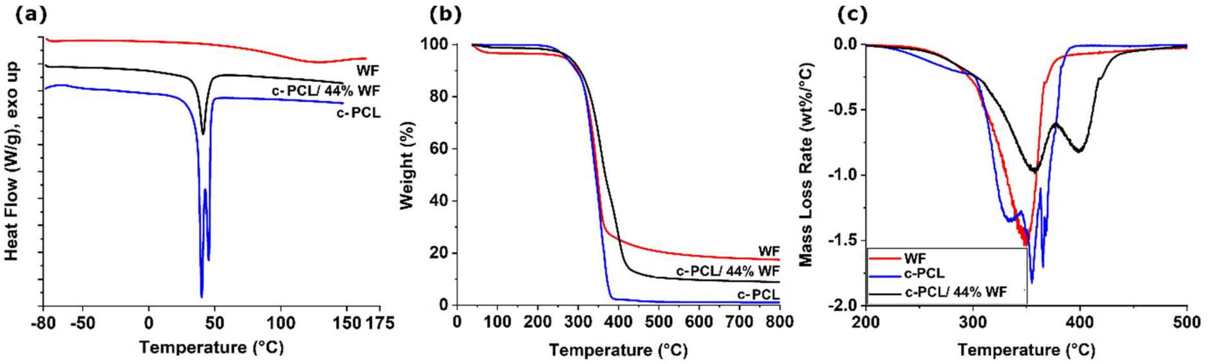

Supplementary Figure 25 Thermal analysis curves of wood fibers (WF), cross-linked polycaprolactone (c-PCL), and c-PCL/ 44% WF including **a)** Differential scanning calorimetry (DSC), **b)** Thermogravimetric analysis (TGA) and **c)** Derivative of thermogravimetric analysis (DTG). DSC curves from heating and cooling cycles of all biocomposites are in the main text.

Supplementary Figure 25 shows the initial weight loss related to moisture evaporation (up to 100 °C) is ca 0.1% for the c-PCL homopolymer, ca 3.4% for wood fibers, and ca 1.3% for c-PCL/ 44% WF, showing the hydrophobicity of the composite. Also, it shows that the degradation of the WF composites is shifted to higher temperatures, and the degradation rate is suppressed.

SEM study of cellulose fibers, MFLC, and the c-PCL biocomposites

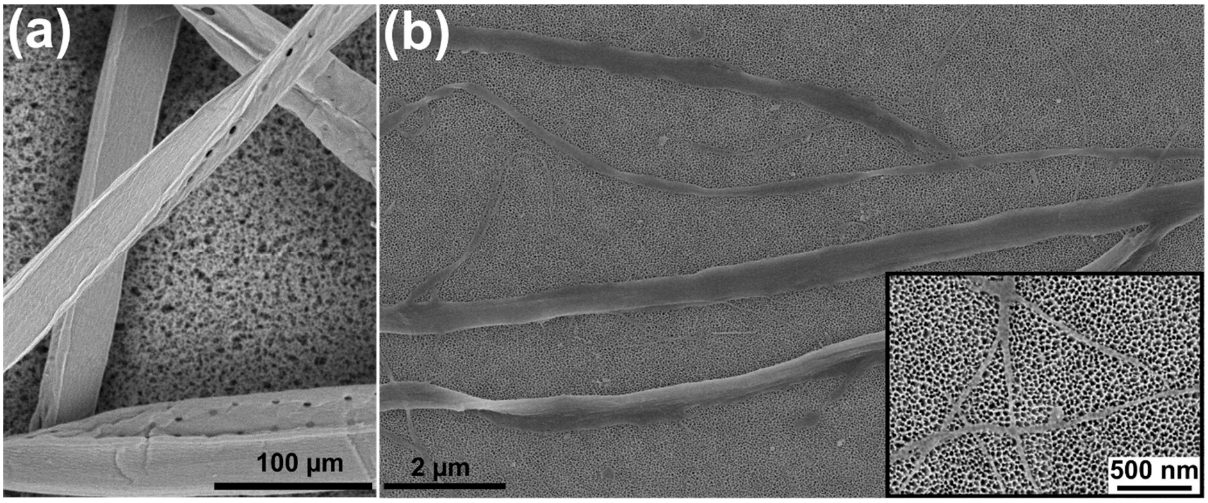

Supplementary Figure 26 Size comparison of wood fibers and microfibrillated lignocellulose (MFLC) **(a)** SEM image of wood fibers. **(b)** SEM images of MFLC.

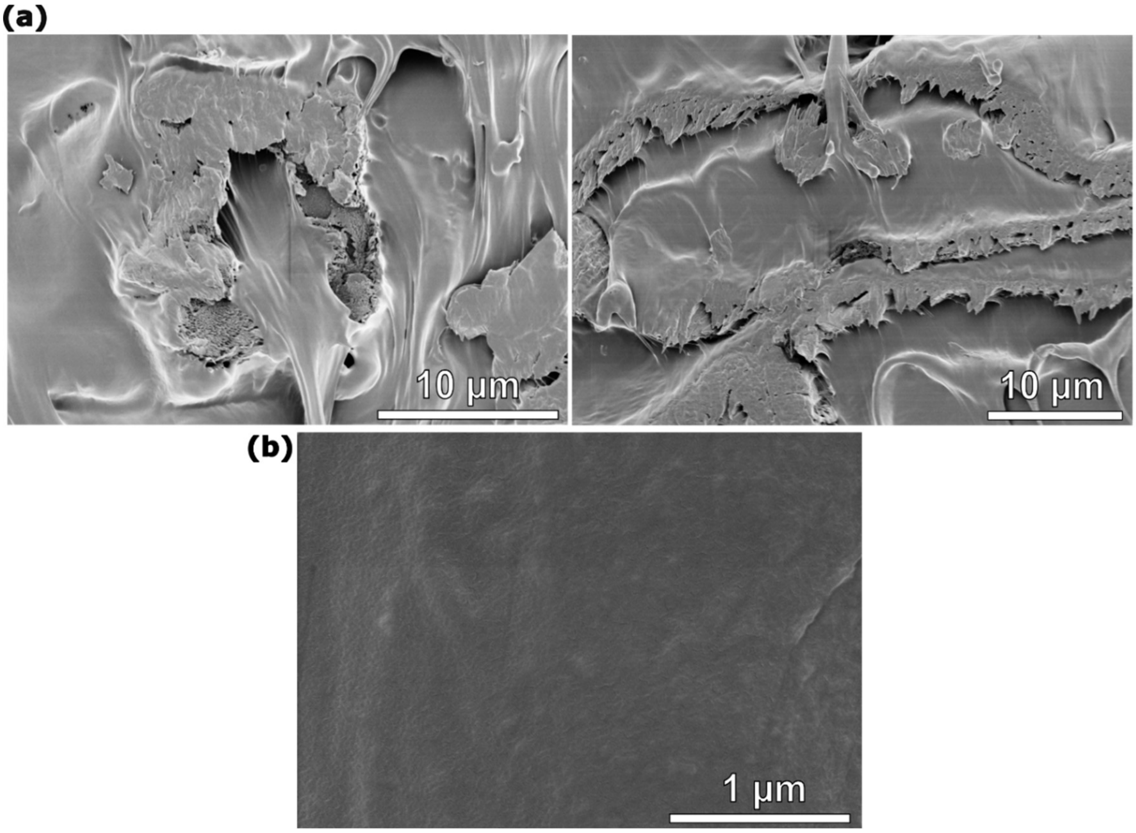

Supplementary Figure 27 Cryo-fracture SEM images of cross-linked polycaprolactone (c-PCL) biocomposite. **(a)** c-PCL/ 25% wood fiber (WF) biocomposite and **(b)** pure c-PCL, showing adhesion of c-PCL to the fiber.

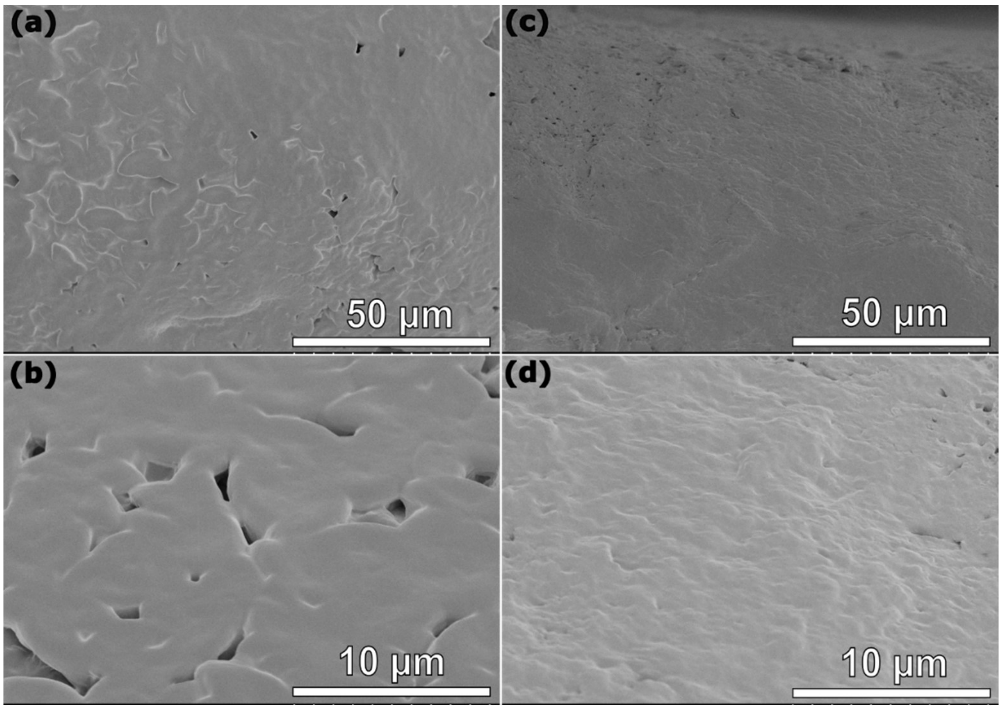

Supplementary Figure 28 SEM images from cross-sections of cross-linked polycaprolactone (c-PCL) biocomposites **a) & b)** 25% wood fiber (WF) and **c) & d)** 27% microfibrillated lignocellulose (MFLC) biocomposites, prepared by UV laser.

Lignin absorbs UV light and generates heat. The composites are reinforced with lignin-containing fibers or fibrils. During cross-section preparation by UV laser, it is very likely that heat generates and melts the polymer matrix. Therefore, the cross-sectional images in Supplementary Figure 28 indicate the well-distribution of polymer matrix in the vicinity of the fibers and lumen region.

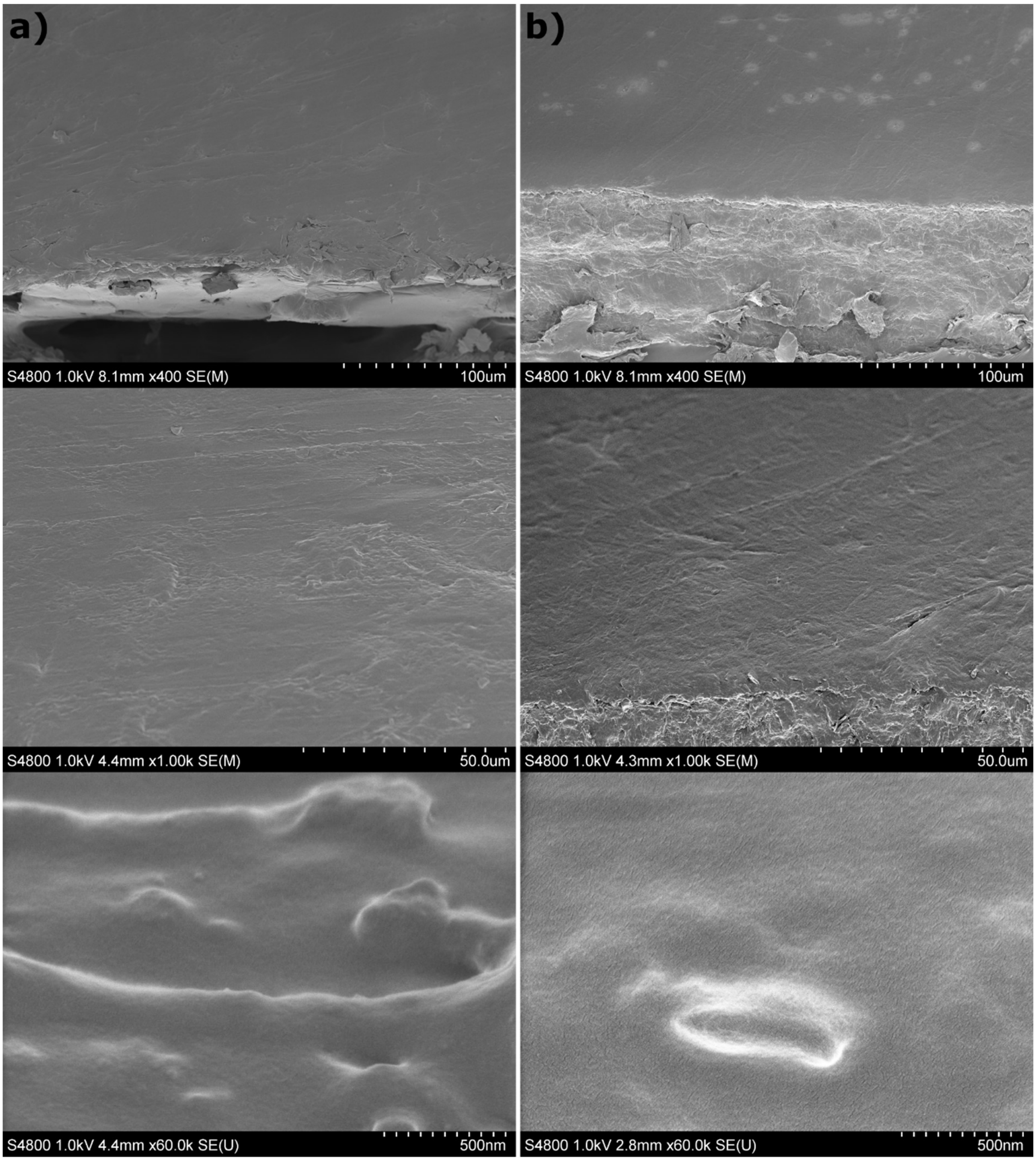

Supplementary Figure 29 Surface SEM images of cross-linked polycaprolactone (c-PCL) biocomposites **a)** c-PCL/WF25 (i.e. 25% wood fiber) and **b)** c-PCL/MFLC27 (i.e. 27% microfibrillated lignocellulose) biocomposites.

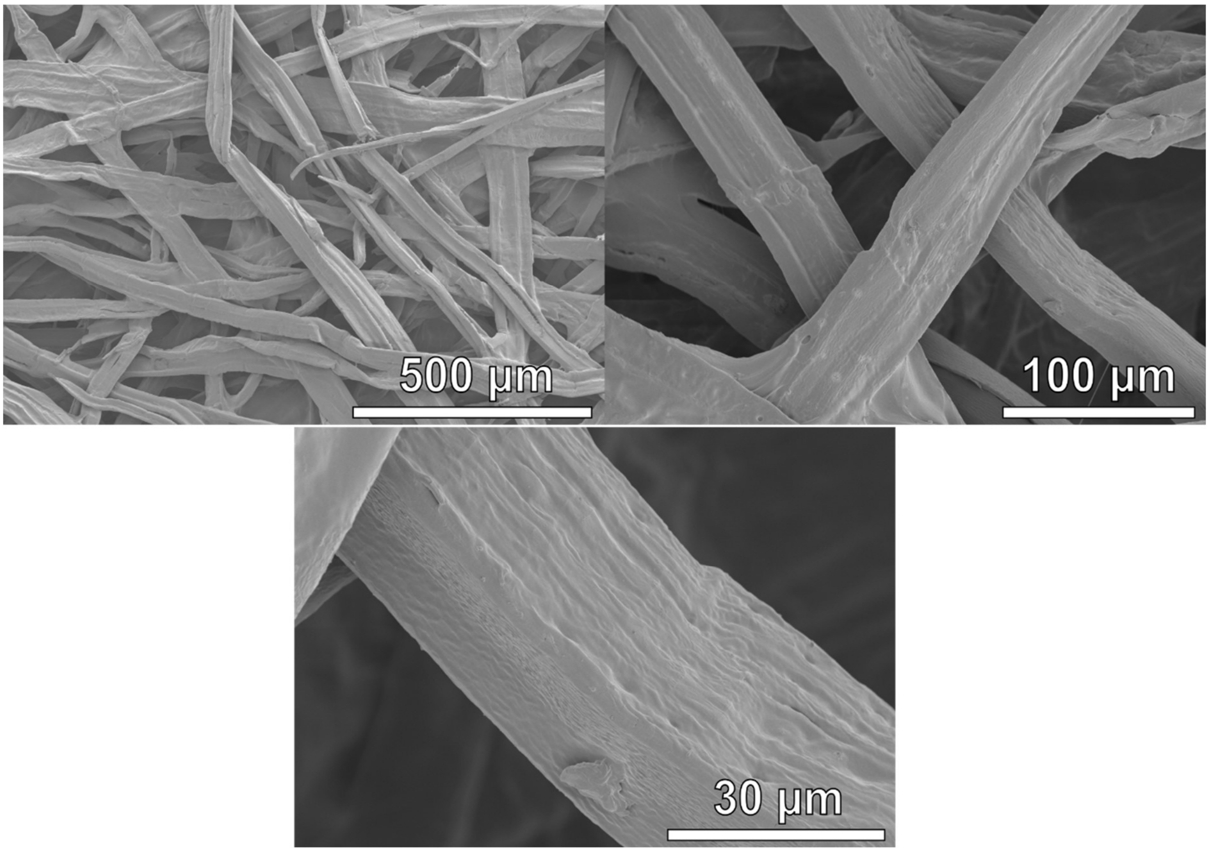

Supplementary Figure 30 Wood fibers extracted from cross-linked polycaprolactone (c-PCL) biocomposites after matrix dissolution and freeze-drying, showing only little damage to the fibers.

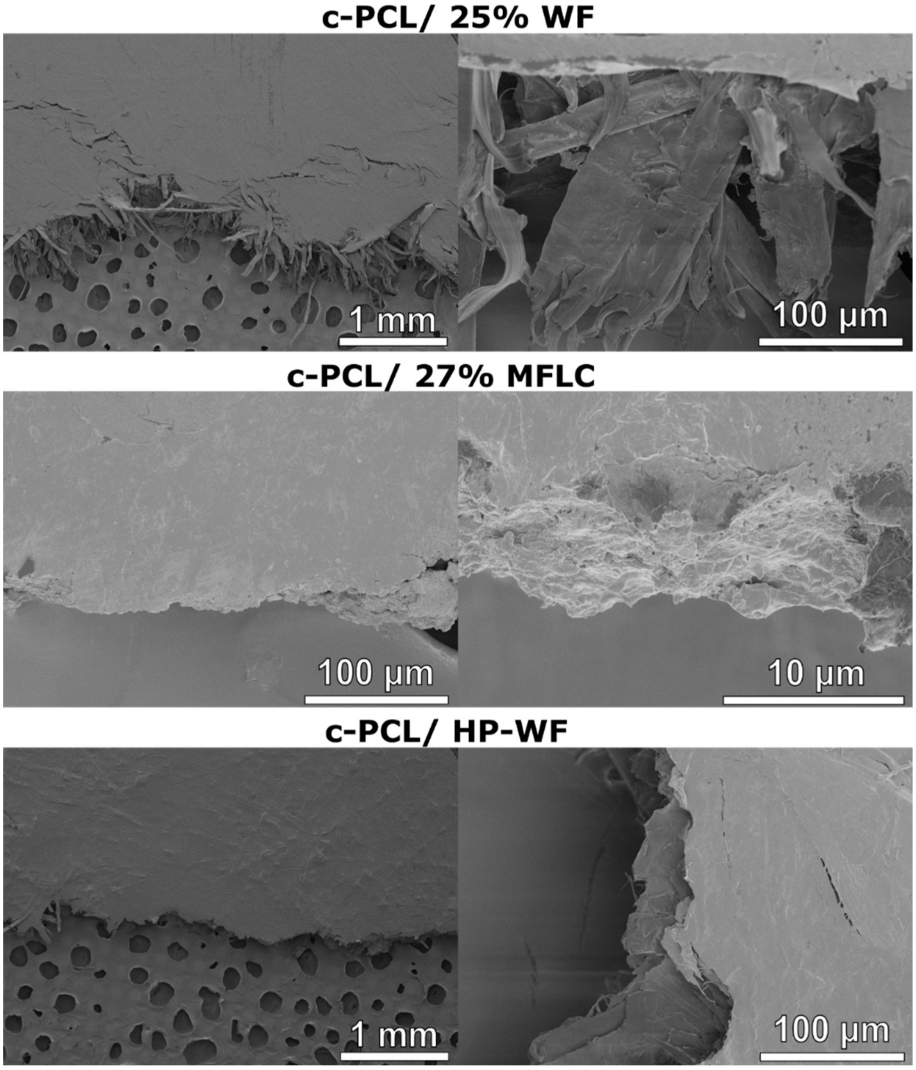

Supplementary Figure 31 Tensile fracture surfaces of wood fiber (WF), microfibrillated lignocellulose (MFLC), and hot-pressed wood fiber (HP-WF) reinforced cross-linked polycaprolactone (c-PCL) biocomposites.

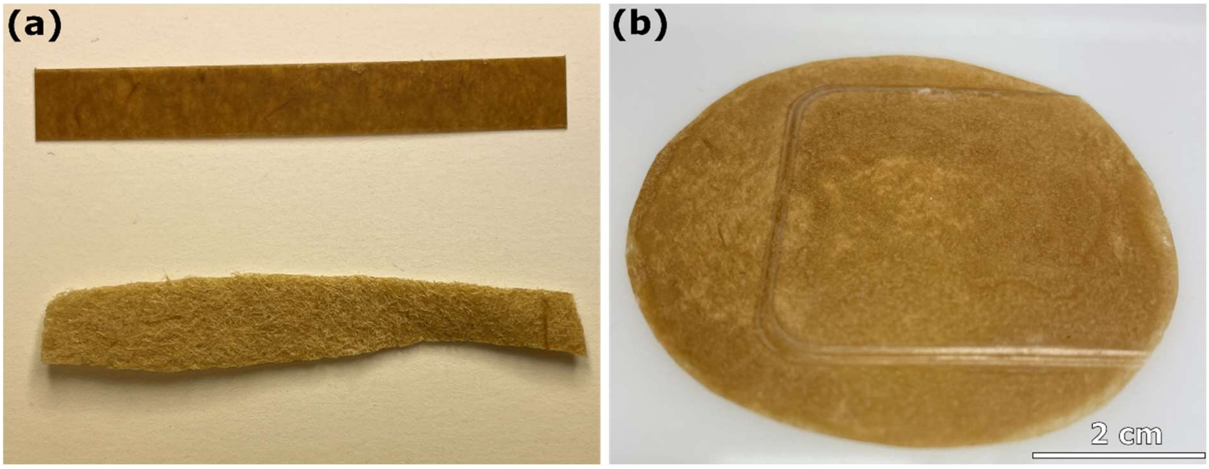

Supplementary Figure 32 Thermal response and thermoformability of cross-linked polycaprolactone (c-PCL) and wood fiber (WF) biocomposites **a)** Illustration of shape memory behavior of WF biocomposites (c-PCL/25wt% WF). (Top image) before, and (bottom image) after exposing to 60 °C (above c-PCL T_m). **b)** Demonstration of 3D re-shaping to wood fiber composite (hot-pressing of flat hot-pressed c-PCL/30 wt% WF into a 3D mold).

As soon as the (hot-pressed) smooth biocomposites exposed to heat, they turned into porous structures (fluffy surface appearance, Supplementary Figure 32a). This appearance is similar to the shape before hot-pressing, Supplementary Figure 32. These characteristics could be important in some applications and during end-of-life management. For instance, it may enhance the composting rate due to the increase of surfaces.

c-PCL biocomposites from hot-pressed wood fibers (HP-WF)

Further information on the preparation of hot-pressed wood fibers is available in refs [4,5].

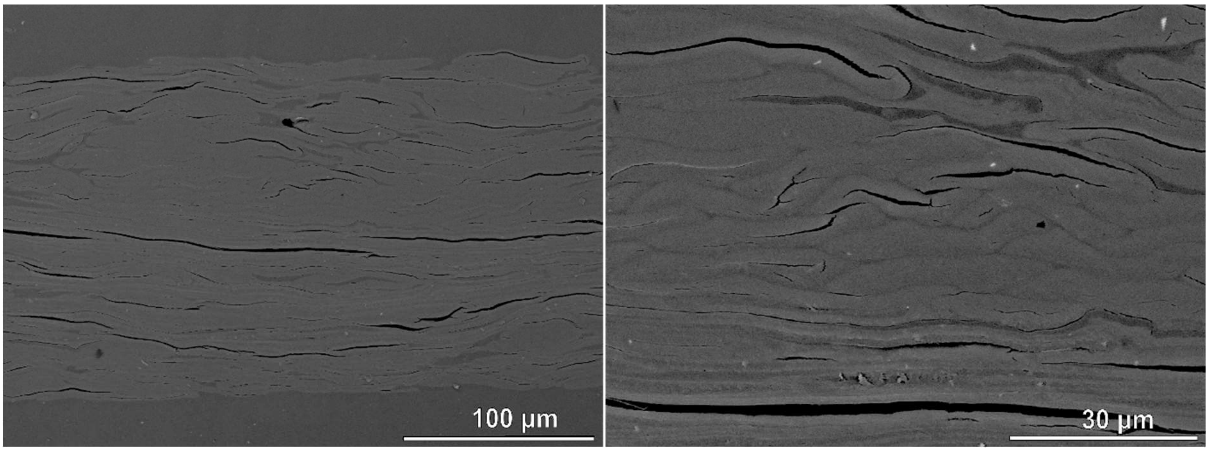

Supplementary Figure 33 SEM images from cross-sectional surfaces of the hot-pressed wood fibers, prepared by epoxy embedding and polishing. Hot-pressed wood fibers reinforcement was later used for biocomposite preparation by in-situ polymerization.

Degree of crystallinity in c-PCL/77% HP-WF is close to pure c-PCL, in contrast to the other biocomposites (Supplementary Table 5). The main reason is possibly the restricted oligomer penetration and nanoconfinement effect during polymerization. The investigation on the structure of this composite shows that although the matrix polymer fills micron-sized gaps between wood fibers, the nano-sized pores within the cell walls remain unfilled (Supplementary Figure 34). Therefore, c-PCL mostly is most likely formed on surfaces and in the micron-sized gaps between fibers, so that degree of crystallinity is less affected by reinforcement.

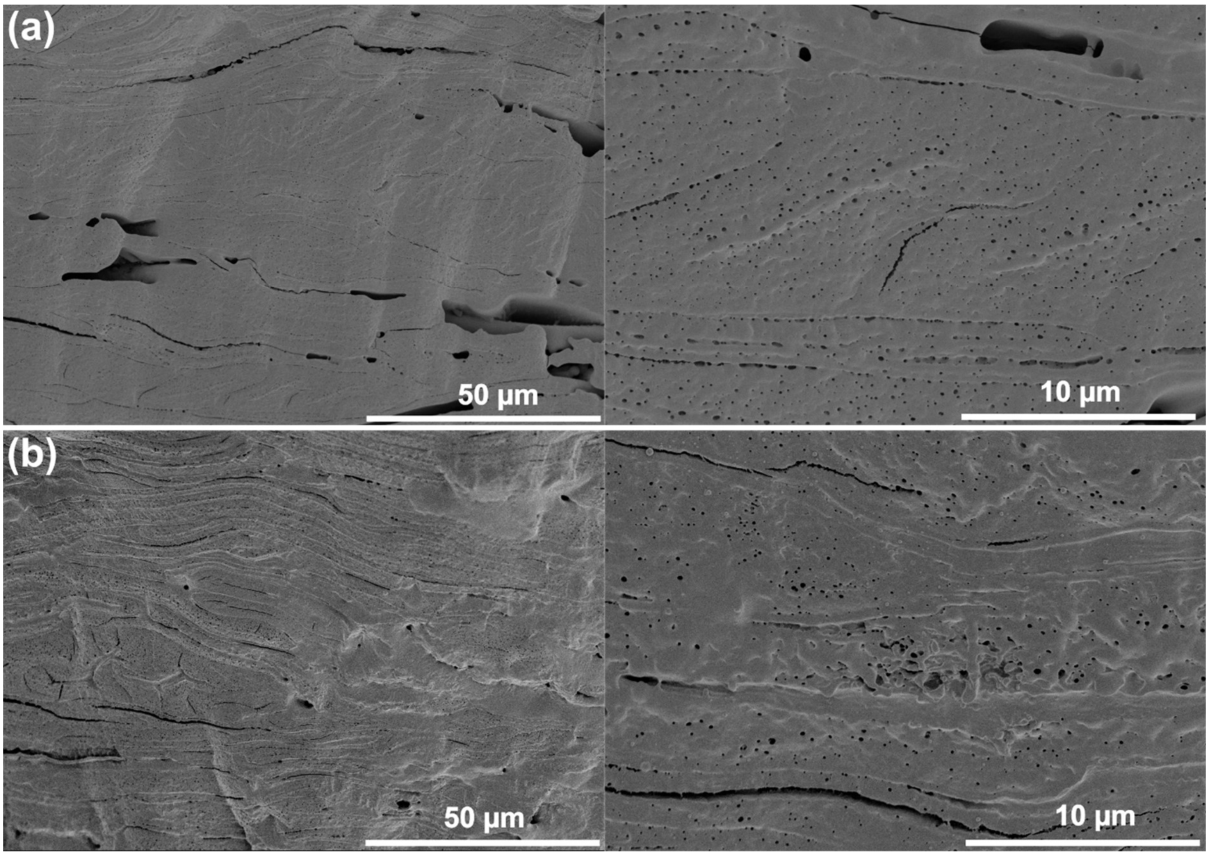

Supplementary Figure 34 Microscopical images of hot-pressed wood fiber biocomposites **(a)** Cross-sectional SEM images of the hot-pressed wood fibers (HP-WF). **(b)** Cross-sectional SEM images of c-PCL/77% HP-WF biocomposite, cut by a UV laser.

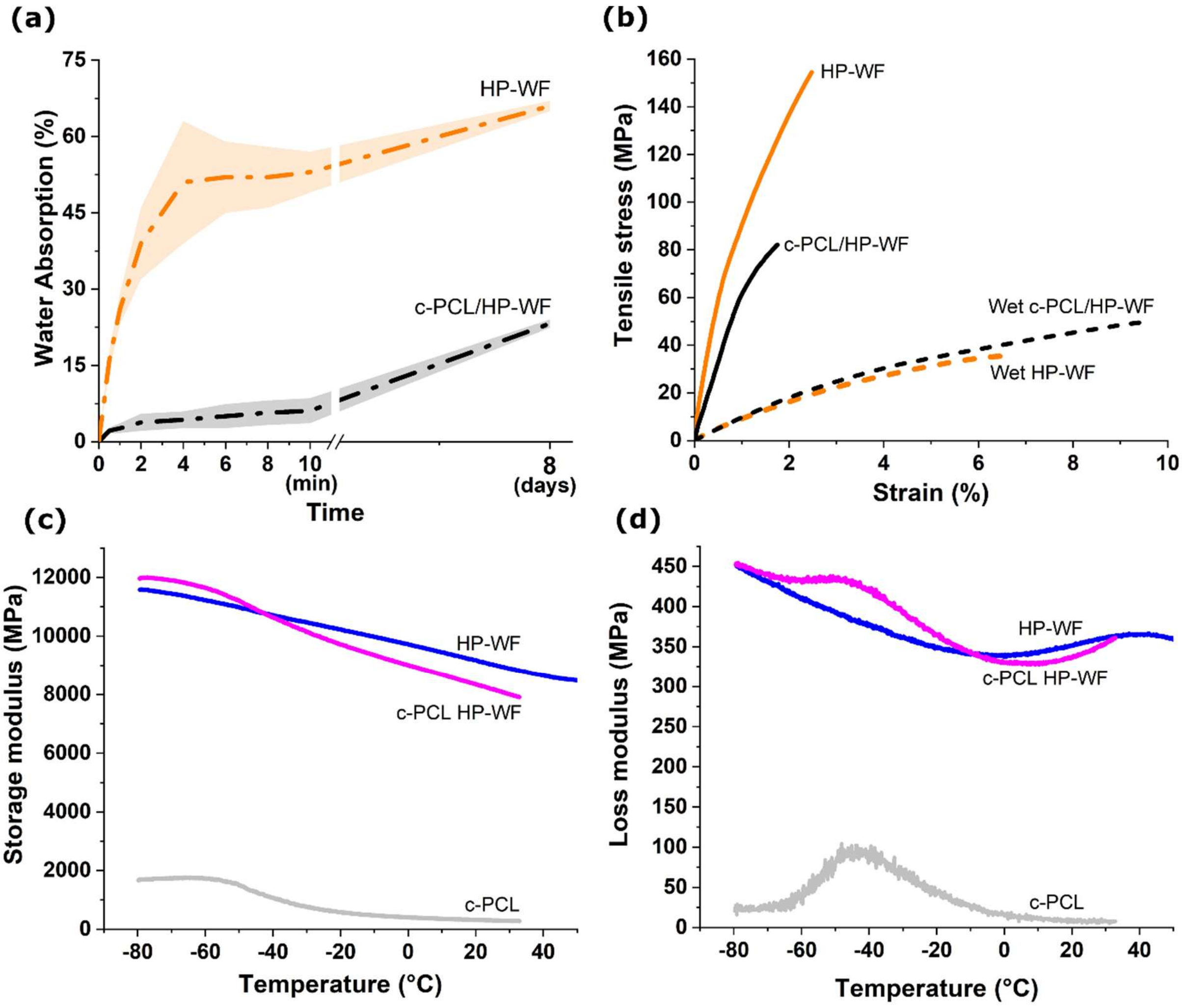

Supplementary Figure 35 Water absorption and mechanical properties of hot-pressed wood fibers (HP-WF), cross-linked polycaprolactone (c-PCL) and c-PCL/(77%)WF biocomposites **(a)** Water absorption, **(b)** dry and wet (after 8 days infiltration in the water) tensile stress-strain curves, **(c)** storage modulus, and **(d)** loss modulus of dry HP-WF and c-PCL/(77%)HP-WF.

The apparent total porosity of HP-WF and c-PCL/HP-WF77 was 23% and 13%, respectively. As a result, the volume fractions of these composites are calculated as: HP-WF (77 v% lignocellulose fiber, 23 v% porosity) and c-PCL/HP-WF77 (67 v% lignocellulose fiber, 20 v% c-PCL, 13 v% porosity). Supplementary Figure 35b shows that the modulus and tensile strength of c-PCL/HP-WF77 are significantly lower than those of HP-WF (6.6 vs 13.2 GPa for modulus, and 82 vs 154 MPa for strength). This reduction in mechanical properties is understandable given that a soft (low modulus) polymer matrix is used. As seen in Supplementary Figure 35, wet mechanical properties of c-PCL/77% HP-WF are very high, showing a modulus as high as 1 GPa, an ultimate strength of ca 50 MPa, and strain at failure of ca 10%. DMA data in Supplementary Figure 35 shows that storage and loss modulus in both hot-pressed wood fibers and hot-pressed wood fiber composite is very high. This is related to high fiber content and hydrophobic c-PCL coverage. The composite shows a clear change in properties around

PCL glass transition. Thermal characteristics of c-PCL/77% HP-WF are calculated and reported in Supplementary Table 5.

Fiber distribution dimensions

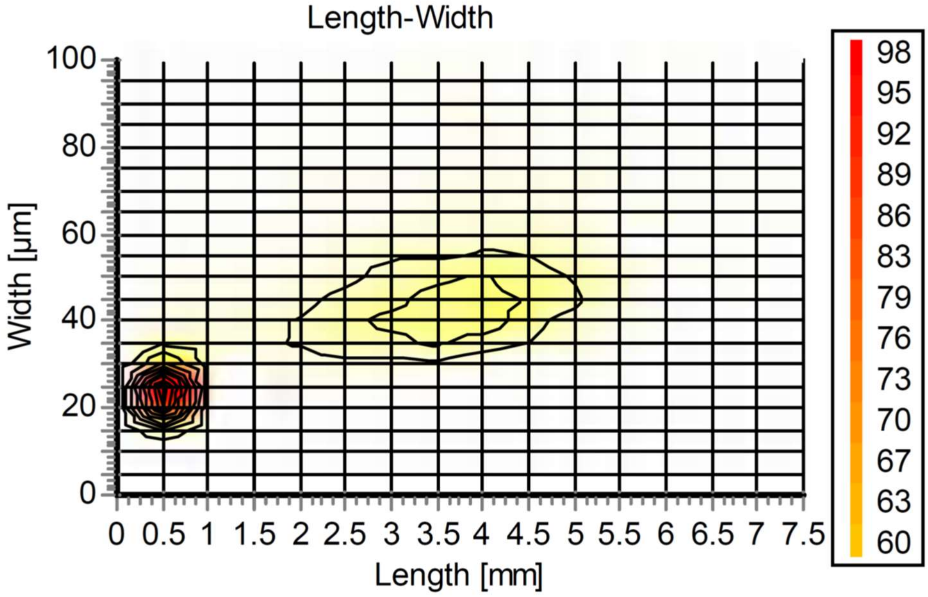

Supplementary Figure 36 Fiber distribution dimensions measured in 20,000 counts using an L&W Fibertester Plus (color shows fiber per mil).

Molten caprolactone oligomer's viscosity

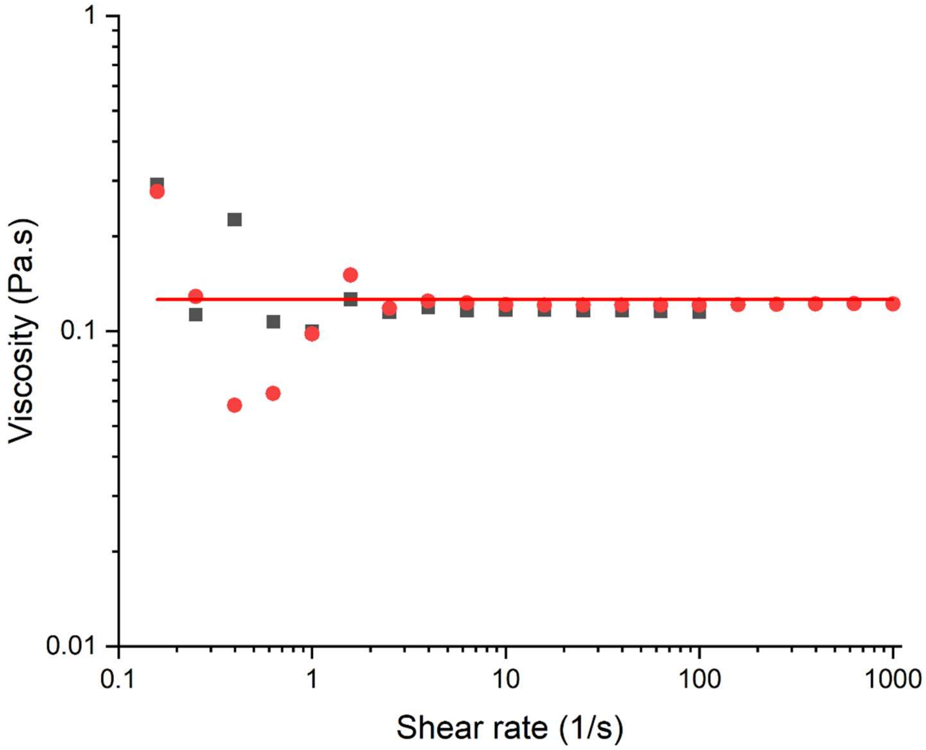

Supplementary Figure 37 Viscosity versus shear rate of caprolactone oligomer at 140 °C, measured using a TA instrument rheometer, parallel plate at 0.5 mm gap in flow sweep. The black and red dots represent two sets of measurements, and the line serves as a visual aid.

Supplementary References:

1 Sheldon, R. A. The E factor 25 years on: the rise of green chemistry and sustainability. *Green Chemistry* **19**, 18-43, doi:10.1039/C6GC02157C (2017).

2 Trost, B. M. The atom economy-a search for synthetic efficiency. *Science* **254**, 1471-1477 (1991).

3 Curzons, A. D., Constable, D. J. C., Mortimer, D. N. & Cunningham, V. L. So you think your process is green, how do you know?—Using principles of sustainability to determine what is green–a corporate perspective. *Green Chemistry* **3**, 1-6, doi:10.1039/B007871I (2001).

4 Oliaei, E. *et al.* Microfibrillated lignocellulose (MFLC) and nanopaper films from unbleached kraft softwood pulp. *Cellulose* **27**, 2325-2341, doi:10.1007/s10570-019-02934-8 (2020).

5 Oliaei, E., Berthold, F., Berglund, L. A. & Lindström, T. Eco-friendly high-strength composites based on hot-pressed lignocellulose microfibrils or fibers. *ACS Sustainable Chemistry & Engineering* **9**, 1899-1910, doi:10.1021/acssuschemeng.0c08498 (2021).
